# Associations Between Geodemographic Factors and Access to Public Health Services Among Chinese Floating Population

**DOI:** 10.3389/fpubh.2020.563180

**Published:** 2020-12-10

**Authors:** Ming Guan

**Affiliations:** ^1^International Issues Center, Xuchang University, Xuchang, China; ^2^Family Issues Center, Xuchang University, Xuchang, China; ^3^School of Business, Xuchang University, Xuchang, China

**Keywords:** floating population, geographical differences, health knowledge, health records, social medical insurance

## Abstract

**Background:** The floating population in urban China is facing multiple barriers to access to comprehensive, affordable, and culturally effective public health services. However, little is known about the role of geodemographic factors. This study aimed to assess the associations between geodemographic factors and access to public health services among the Chinese floating population.

**Methods:** This study employed the data from the 2015 Migrant Dynamic Monitoring Survey data in China. Descriptive statistical analysis and principal component analysis were used to provide basic characteristics of the main variables. Multiple logistic models were used to analyze how province-level units, economic regions, and economic zones had significant associations with residential health records establishment, social medical insurance, and types and methods of health knowledge attainment in urban China. Using multiple indicator multiple cause models, the association between geodemographic factors and types and methods of health knowledge attainment was studied.

**Results:** The results indicated that there was regional unbalance in the case of residential health records. Regional differences were significantly associated with social medical insurance. Provincial differences were significantly associated with health knowledge attainment. There were regional differences in the methods of health knowledge attainment. In the most provincial units, geodemographic factors had significant associations with types and methods of health knowledge attainment.

**Conclusions:** This study confirmed empirical associations between geodemographic factors and access to public health services among Chinese floating population. The relevant suggestion was that provincial units with less-developed public health services should enhance their capabilities to equalize public health services.

## What Is Already Known?

- Health status was poor among floating population in China.

## What are the New Findings?

- Health records among floating population were geographically distributed unevenly in China.
- Social medical insurance among the floating population was geographically covered unevenly in China.
- Health knowledge among the floating population was geographically obtained unevenly in China.

## What do the New Findings Imply?

- There is an urgent need to balance health service utilization for the floating population in China.
- Findings from this study have the potential to redesign health policy for the floating population in China.

## Introduction

Since entry into the new century, the Chinese floating population, especially the interprovincial floating population (1), increase year by year due to rapid economic growth. The floating population referred to the large and increasing number of migrants without local household registration status and had become a new demographic phenomenon in China. Most of these migrants move from the rural areas of the central and western parts of China to the eastern and coastal metropolitan areas in pursuit of a better life.

Recent evidence suggested that the floating population in China was characterized by stigma and discrimination (2), poor psychological and social health (3), poor sense of belonging (4), and high prevalence of induced abortion (5). It has previously been observed that they exhibited poor awareness of reproductive health (6) and lived in poor living environment (7). Although the prevalence of diabetes in the floating population was lower than that in the general population, the potential threat brought by the diabetes in floating population was imponderable due to the growing population number and the poor disease management (8). Despite low percentage of participation in moderate and vigorous activities (9), prevalent drinking behavior (10), and smoking behaviors (11) were high among them. Since access to public health services affected the medical treatment seeking behaviors of floating population (12–14), geodemographic factors could be speculated to have association with their health status.

Early research reported the uneven supply of public health services across migrant population groups and regions. For instance, the utilization of health services among young migrants (15) and old-aged migrants (16) were insufficient in comparison with the general population. It was impossible to cover internal migrants with lower socioeconomic status and across provinces by public health services (17). Even worse, there was an imbalance of basic public health service utilization between urban and suburban areas (18). Thus, the relationship between geodemographic factors and access to public health services possibly existed among the Chinese floating population.

This study aimed to assess equalization of basic public health services geographically. With an official publicly available survey data, the present study tried to investigate the associations between geodemographic factors and access to public health services among internal migrants in China. The statistical outcomes would reveal the targeted relationships.

## Methods

### Data

This study used data from 2015 Migrant Dynamic Monitoring Survey. The survey adopted a stratified three-stage probability proportionate to size sampling, and the annual national data on migrants from each province in 2014 was considered the basic sampling frame. According to the random principle through the multistage clustering sampling method, the sample of the floating population was collected from 6 major industries in 170 counties and districts from 31 province-level units and Xinjiang Production and Construction Corps (XPCC). The subject of the survey was the inflow of persons aged 15 and above who lived in the place of inflow for more than 1 month and were not registered in the district (county, city). The sampling method of this data was based on the annual report data of floating population, all staff in 31 provinces (districts and municipalities) and XPCC in 2014. A total sample size of 206,000 was projected for the survey, involving a total of approximately 500,000 floating population family members. Here, only the part of the migrant population that began to move after 1978 was studied. Thus, in the total sample, persons were excluded due to marriages, residential mobility, seeking help from relatives and friends, accepting training, military service, and giving birth. And, the duration of population floating was constrained to 1978–2015.

### Main Variables

Establishment of health records was reflected by the question: “Do you have established health records in your local community?” The response options were “no, I have never heard of this,” “no, but I have heard of this,” “I have established health records,” and “I don't know.” The “no” response was coded as 0, the “yes” response was coded as 1, and The “I don't know” response was coded as missing values.

Social medical insurance was reflected by the question: “Which type of social medical insurance were you covered by” The six choices were the following: new rural cooperative medical scheme (NRCMS), Cooperative medical insurance for urban and rural residents (CMIURR), urban resident based basic medical insurance (URBMI), urban employee-based basic medical insurance (UEBMI), and free medical treatment (FMT).

The methods of access to health knowledge were reflected by a multiple choice question: “How did you access health knowledge?” The eight choices were the following: lecture (1 = yes, 0 = no), books/magazine/CD (1 = yes, 0 = no), radio/TV programmes (1 = yes, 0 = no), face-to-face consultation (1 = yes, 0 = no), online medical consultation (1 = yes, 0 = no), community advocacy (1 = yes, 0 = no), bulletin board(1 = yes, 0 = no), SMS/WeChat (1 = yes, 0 = no). For convenience, the methods of access to health knowledge were abbreviated by knpatha, knpathb, knpathc, knpathd, knpathe, knpathf, knpathg, and knpathh, respectively.

The types of access to health knowledge were reflected by a multiple-choice question: “Did you access the below health knowledge in the destines in the last year?” The response options were occupational disease prevention (1 = yes, 0 = no), nutritional health knowledge (1 = yes, 0 = no), reproduction and contraception/eugenics (1 = yes, 0 = no), chronic disease prevention (1 = yes, 0 = no), smoking control (1 = yes, 0 = no), mental disorders (1 = yes, 0 = no), tuberculosis (1 = yes, 0 = no), STD/AIDS (1 = yes, 0 = no), and other infectious diseases(1 = yes, 0 = no). For convenience, the types of access to health knowledge were abbreviated by kntypea, kntypeb, kntypec, kntyped, kntypee, kntypef, kntypeg, kntypeh, and kntypei, respectively.

The coverage of public health services was reflected by establishment rate of health records (ERHR), social medical insurance ratio (SMIR), inaccess health knowledge ratio (IHKR), and methods of inaccess to health knowledge ratio (MIHKR). The ERHR (=persons with health records/total sample), SMIR (=persons with social medical insurance/ total sample), IHKR (=persons with inaccess to health knowledge/total sample), and MIHKR (=persons with inaccess methods of access to health knowledge/total sample) can be calculated with formulas.

### Statistical Strategies

First, a descriptive analysis was conducted to analyze socioeconomic factors by chi-square test. Frequency and percentage were employed to reflect categories' distribution in the socioeconomic variables. Second, the logistic regression was used to reflect geographic equality of public health services in the province-level units, economic regions, and economic zones. Thus, public health services like NRCMS (1 = yes, 0 = no), CMIURR (1 = yes, 0 = no), URBMI (1 = yes, 0 = no), UEBMI (1 = yes, 0 = no), and FMT (1 = yes, 0 = no) were outcome variables. And geographic variables were independent variables. When regressing economic regions, Pearl River Delta was referred. This was because Pearl River Delta attracted a number of the floating population under the increasing regional gaps and global economic bloom. From 2000 to 2010, the floating population mainly flowed into three economic regions and provincial capitals and cities in the eastern China. Third, the multiple indicator multiple cause (MIMIC) model was adopted to measure the associations between socio-economic factors and types of access to health knowledge and methods in a specific province-level unit. The socio-economic factors were age “(2015+9/12) minus (birth year+ birth month /12)”, gender (male = 1, female = 0), education (educated = 1, uneducated = 0), Hukou (household registration, rural = 1, other = 0), marital status (first married = 1, others = 0), income (unit: Chinese Yuan), and flowing direction (interprovince = 1, other = 0) were the main covariates. Principal component analysis (PCA) in conjunction with varimax rotation with Kaiser's criterion was performed to explore the structure of types and methods of access to health knowledge. The Kaiser–Meyer–Olkin (KMO>0.7, adequate) coefficient and Bartlett's test would be measured to reflect suitable to conduct PCA. The internal consistency would be assessed by Cronbach's alpha. In the confirmatory factor analysis, root mean square error of approximation (RMSEA: <0.05 close, < 0.08 good, <0.10 reasonable) was employed to measure goodness-of-fit of MIMIC models.

### Institutional Review Board (IRB) Approval

Regarding ethical acceptability, this study used secondary data from the 2015 Migrant Dynamic Monitoring Survey and was deemed excluded from IRB review. With respect to human rights, this study implemented to protect human subjects with the anonymous survey dataset.

## Results

### Sample Characteristics

Table 1 showed that most of the sample accepted middle school and above education. Regarding Hukou, more than 85% of the floating population had agricultural Hukou. Regarding marital status, 80% of them were first married, followed by 16% unmarried. More than half of them were interprovincial migrants. Number of males surpassed number of females. Regarding age, mean male age was somewhat higher than that of females. Most of the floating population were adult laborers who were younger than 60 years old and earned <10 thousand Chinese Yuan per month in the last year.

**Table 1:** Sample characteristics (total = 165,709).

	**Frequency**	**percentage**
**Age**		
15–35	87,885	53.04
36–59	75,744	45.71
60 or above	2,080	1.26
**Gender**		
Female	70,345	42.45
Male	95,364	57.55
**Education attainment**		
Illiterate	2,744	1.66
Elementary school	22,740	13.72
Primary school	89,229	53.85
High school	35,776	21.59
Junior college	10,294	6.21
Undergraduate	4,643	2.80
Graduate	283	0.17
**Hukou types**		
Agricultural Hukou	142,314	85.88
Non-Agricultural Hukou	21,202	12.79
Resident transferred from agricultural Hukou	1,917	1.16
Resident transferred from non-agricultural Hukou	276	0.17
**Marital status**		
Unmarried	26,483	15.98
First married	132,569	80.00
Remarried	2,482	1.50
Divorced	3,334	2.01
Widowed	841	0.51
**Income**		
0–9,999	145,724	87.94
10,000–49,999	19,494	11.76
50000 or more	491	0.30
**Flowing direction**		
Interprovince	86,412	52.15
Intercity	48,314	29.16
Intercounty	30,962	18.68
International	21	0.01

**Figure 1:**
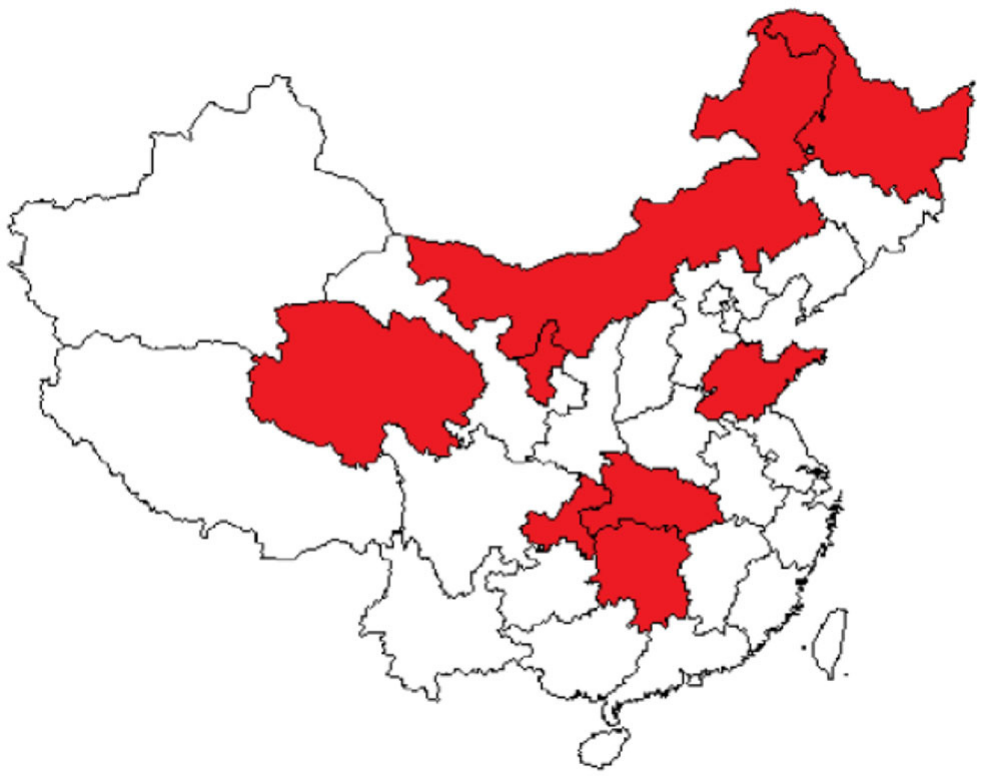
Province-level units ERHR>40%.

**Figure 2:**
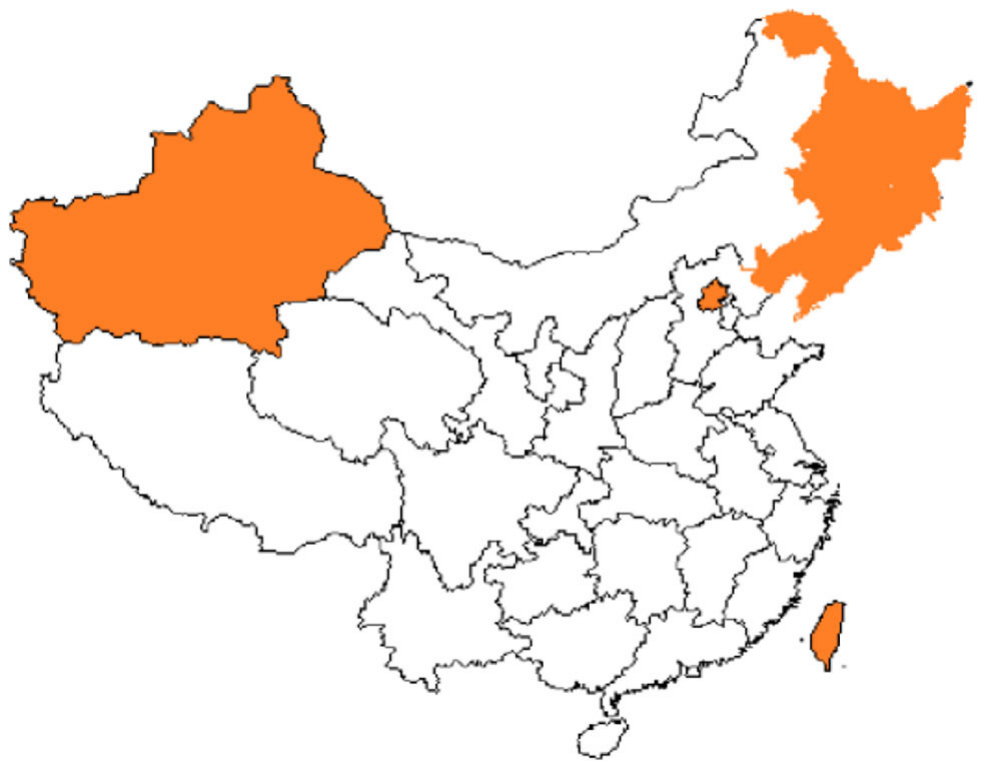
Province-level units with SMIR >10%.

**Figure 3:**
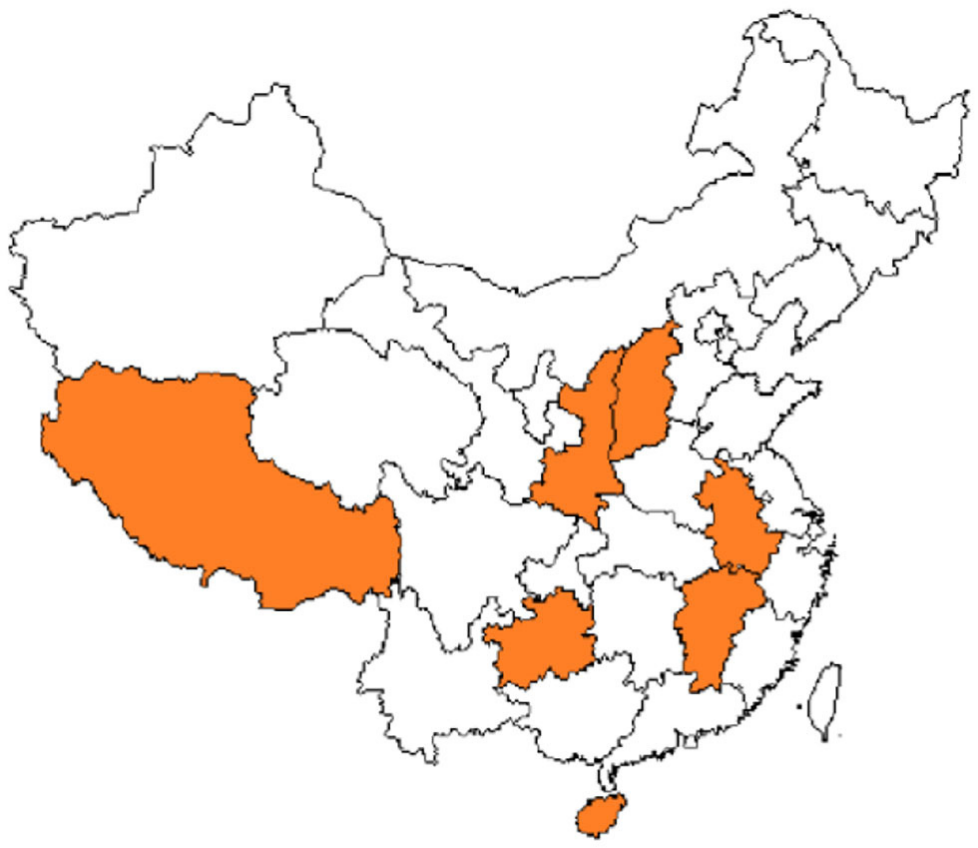
Province-level units with IHKR>10%.

**Figure 4:**
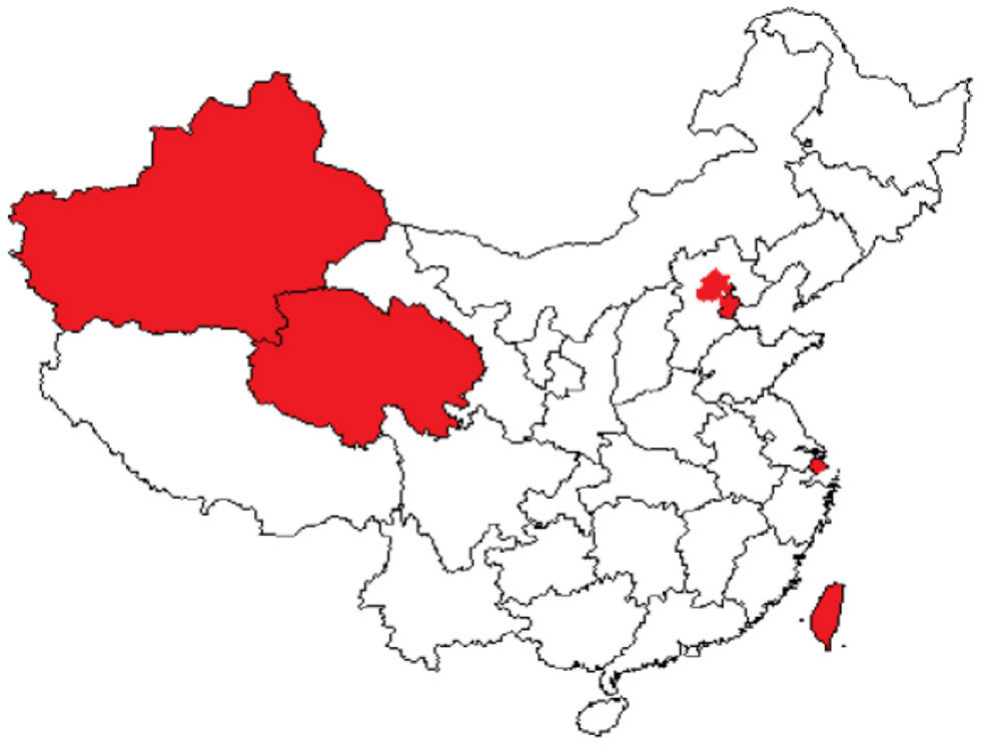
Province-level units with MIHKR >1.

The coverage rate of social medical insurance reached 93.66%. See Figure 1, provinces with establishment rate of floating population health records>40% were Inner Mongolia (40.238%), Heilongjiang (52.725%), Shandong (47.535%), Hubei (50.612%), Hunan (45.419%), Chongqing (40.756%), Qinghai (50.431%), and Ningxia (47.793%). Figure 2 showed provinces with uncoverage rates of social medical insurance>10% were Beijing (14%), Liaoning (13%), Jilin (12%), Heilongjiang (16%), Xinjiang (12%), XPCC (14%), Taiwan (20%), and Hongkong (17%). See Figure 3, provinces with unaccess to health knowledge>10% were Shanxi (10%), Anhui (11%), Jiangxi (12%), Hainan (14%), Guizhou (11%), Tibet (21%), and Shannxi (10%). Figure 4 showed province-level units with more than one knowledge access method were Beijing, Tianjin, Inner Mongolia, Shanghai, Qinghai, XPCC, and Taiwan.

See Table 2. More than 30% of the floating population had established health records. The establishment rate of health records of males was higher than that of females. Regarding social medical insurance, there were significant gender difference in NRCMS (new rural cooperative medical scheme), CMIURR (cooperative medical insurance for urban and rural residents), URBMI (urban resident based basic medical insurance), and UEBMI (urban employee-based basic medical insurance). Simultaneously, the coverage rate of social security programs of males was higher than that of females. Regarding access to health knowledge, there were significant gender differences in kntypea (access to occupational disease prevention knowledge), kntypeb (access to nutritional health knowledge), kntypec (access to reproduction and contraception/eugenics knowledge), kntypee (access to smoking control knowledge), kntypef (access to mental disorders knowledge), kntypeh (access to STD/AIDS knowledge), and kntypei (access to other infectious diseases knowledge). Likewise, access to knowledge rate of male was higher than that of females. Regarding methods of access to health knowledge, there were significant gender difference in knpatha (access to health knowledge with lecture), knpathd (access to health knowledge with face-to-face consultation), knpathe (access to health knowledge with online medical consultation), knpathf (access to health knowledge with community advocacy), and knpathg (access to health knowledge with bulletin board). Similarly, methods of access to health knowledge of males were more than those of females.

**Table 2:** Gender difference in the access to public health services.

	**Total**	**Male (%)**	**Female (%)**	**Chi-square**	***P***	**Significance**
Health records	138,336			38.3974	0.000	^***^
No		38.13	27.76			
Yes		19.14	14.96			
**Social medical insurance**						
NRCMS	113,776			5.8539	0.016	^**^
No		17.02	12.90			
Yes		40.32	29.76			
CMIURR	161,867			19.1787	0.000	^***^
No		55.07	40.74			
Yes		2.30	1.89			
URBMI	161,917			14.6320	0.000	^***^
No		54.43	40.26			
Yes		2.94	2.37			
UEBMI	162,168			45.2077	0.000	^***^
No		47.14	35.58			
Yes		10.22	7.05			
FMT	161,843			2.6413	0.104	
No		57.32	42.60			
Yes		0.05	0.03			
**Types of access to health knowledge**						
Kntypea	165,700			147.3525	0.000	^***^
No		33.49	25.96			
Yes		24.06	16.49			
Kntypeb	165,700			523.8908	0.000	^***^
No		21.88	13.83			
Yes		35.67	28.63			
Kntypec	165,700			3.0e+03	0.000	^***^
No		22.07	10.88			
Yes		35.48	31.57			
Kntyped	165,700			0.0085	0.927	
No		33.58	24.76			
Yes		23.97	17.69			
Kntypee	165,700			2.6e + 03	0.000	^***^
No		19.04	19.29			
Yes		38.51	23.16			
Kntypef	165,700			32.9167	0.000	^***^
No		46.82	34.06			
Yes		10.72	8.39			
Kntypeg	165,700			1.1763	0.278	
No		35.99	26.66			
Yes		21.56	15.79			
Kntypeh	165,700			63.9897	0.000	^***^
No		25.48	17.96			
Yes		32.06	24.49			
Kntypei	165,700			25.7292	0.000	^***^
No		36.73	26.58			
Yes		20.82	15.87			
**Methods of access to health knowledge**						
Knpatha	152,342			104.4956	0.000	^***^
No		40.62	29.39			
Yes		16.55	13.44			
Knpathb	152,342			2.0834	0.149	
No		32.58	24.56			
Yes		24.60	18.26			
Knpathc	152,342			0.3580	0.550	
No		11.67	8.80			
Yes		45.50	34.03			
Knpathd	152,342			189.6574	0.000	^***^
No		42.50	30.47			
Yes		14.68	12.35			
Knpathe	152,342			0.1020	0.749	
No		33.46	25.03			
Yes		23.72	17.80			
Knpathf	152,342			64.1384	0.000	^***^
No		35.71	25.88			
Yes		21.47	16.94			
Knpathg	152,342			13.3856	0.000	^***^
No		9.49	6.81			
Yes		47.69	36.02			
Knpathh	152,342			1.4522	0.228	
No		24.89	18.77			
Yes		32.29	24.05			

*Standard deviations in parentheses*.

*** and

** *represent 1 and 5%*.

### Association Between Province-Level Units and Types of Access to Health Knowledge

See Table 3. Shanxi, Inner Mongolia, Hainan, Tibet, Gansu, and Xinjiang had significant associations with access to kntypea (access to occupational disease prevention knowledge). Hebei, Shanxi, Inner Mongolia, Anhui, Jiangxi, Henan, Guangdong, Guangxi, Hainan, Sichuan, Guizhou, Yunan, Tibet, Shannxi, Gansu, Qinghai, Ningxia, and Xinjiang had significant associations with kntypeb (access to nutritional health knowledge). Fujian, Shandong, Hubei, Hunan, Guangdong, Guangxi, Chongqing, Yunan, Tibet, Ningxia, and Xinjiang had significant associations with kntypec (access to reproduction and contraception/eugenics knowledge). Hebei, Shanxi, Hainan, Tibet, and XPCC had significant associations with kntyped (access to chronic disease prevention knowledge). Tibet had significant associations with kntypee (access to smoking control knowledge). Shanghai, Fujian, Hubei, Hunan, Guangdong, Guangxi, Chongqing, Sichuan, and Beijing had significant associations with kntypef (access to mental disorders knowledge). Hebei, Hunan, Chongqing, Sichuan, Guizhou, Yunan, Tibet, Gansu, Qinghai, Ningxia, Xinjiang, XPCC, and Beijing had significant associations with kntypeg (access to tuberculosis knowledge). Fujian, Jiangxi, Henan, Hubei, Hunan, Guangdong, Guangxi, Hainan, Chongqing, Sichuan, Guizhou, Yunan, Tibet, Gansu, Qinghai, Xinjiang, and XPCC had significant associations with kntypeh (access to STD/AIDS knowledge). Shanghai, Fujian, Henan, Hubei, Hunan, Guangdong, Guangxi, Chongqing, Sichuan, Guizhou, Yunan, Gansu, Qinghai, Ningxia, Xinjiang, XPCC, and Beijing had significant associations with kntypei (access to other infectious diseases knowledge). But, Tianjin, Liaoning, Jilin, Heilongjiang, Jiangsu, and Zhejiang had no significant associations with kntypea (access to occupational disease prevention knowledge), kntypeb (access to nutritional health knowledge), kntypec (access to reproduction and contraception/eugenics knowledge), kntyped (access to chronic disease prevention knowledge), kntypee (access to smoking control knowledge), kntypef (access to mental disorders knowledge), kntypeg (access to tuberculosis knowledge), kntypeh (access to STD/AIDS knowledge), and kntypei (access to other infectious diseases knowledge). Thus, the access imbalance of health knowledge types was reflected by the associations between province-level units and types of access to health knowledge.

**Table 3:** Associations between province-level units and types of access to health knowledge (sample = 165,649).

	**Kntypea**	**Kntypeb**	**Kntypec**	**Kntyped**	**Kntypee**	**Kntypef**	**Kntypeg**	**Kntypeh**	**Kntypei**
Tianjin	0.982 (0.321)	0.764 (0.284)	0.993 (0.328)	0.923 (0.306)	0.817 (0.270)	1.712 (0.826)	1.205 (0.421)	1.093 (0.358)	1.677 (0.603)
Hebei	0.659 (0.168)	0.524^**^ (0.156)	0.846 (0.218)	0.630^*^ (0.163)	0.763 (0.198)	0.968 (0.390)	0.592^*^ (0.164)	0.760 (0.195)	1.031 (0.301)
Shanxi	0.541^**^ (0.139)	0.501^**^ (0.150)	1.245 (0.324)	0.558^**^ (0.145)	0.972 (0.254)	1.143 (0.463)	0.718 (0.200)	1.054 (0.272)	0.935 (0.274)
Inner Mongolia	0.500^***^ (0.129)	0.470 ^**^ (0.140)	1.179 (0.306)	0.889 (0.231)	1.190 (0.311)	1.384 (0.559)	1.199 (0.333)	1.036 (0.267)	1.091 (0.319)
Liaoning	0.762 (0.196)	0.652 (0.195)	0.934 (0.244)	0.923 (0.241)	1.085 (0.285)	1.437 (0.582)	1.093 (0.305)	1.102 (0.285)	1.207 (0.355)
Jilin	0.697 (0.179)	0.673 (0.201)	1.177 (0.306)	1.165 (0.302)	1.249 (0.326)	1.912 (0.772)	1.385 (0.385)	1.345 (0.346)	1.498 (0.438)
Heilongjiang	0.665 (0.170)	0.658 (0.196)	1.207 (0.312)	1.172 (0.303)	1.091 (0.284)	1.619 (0.652)	1.386 (0.384)	1.195 (0.306)	1.452 (0.423)
Shanghai	1.097 (0.407)	1.436 (0.658)	1.368 (0.524)	1.544 (0.576)	1.094 (0.415)	2.946^**^ (1.481)	1.293 (0.508)	1.171 (0.435)	2.066^*^ (0.824)
Jiangsu	0.990 (0.253)	0.741 (0.221)	1.243 (0.322)	1.141 (0.295)	1.213 (0.316)	1.899 (0.765)	1.238 (0.343)	1.371 (0.352)	1.475 (0.430)
Zhejiang	0.656 (0.168)	0.662 (0.200)	10.379 (0.358)	0.969 (0.251)	10.167 (0.305)	1.866 (0.753)	1.110 (0.308)	1.519 (0.391)	1.59 (0.465)
Anhui	0.660 (0.168)	0.558^**^ (0.166)	1.375 (0.355)	0.833 (0.215)	0.933 (0.242)	1.572 (0.632)	0.975 (0.269)	1.209 (0.309)	1.301 (0.378)
Fujian	0.836 (0.214)	0.763 (0.228)	2.142^***^ (0.556)	0.963 (0.250)	1.462 (0.381)	2.181^*^ (0.879)	1.328 (0.368)	2.295^***^ (0.590)	1.743^*^ (0.509)
Jiangxi	0.733 (0.187)	0.524 ^**^ (0.156)	1.329 (0.344)	0.798 (0.206)	0.953 (0.248)	1.724 (0.694)	1.085 (0.300)	1.537^*^ (0.394)	1.483 (0.432)
Shandong	0.867 (0.222)	0.772 (0.230)	2.489^***^ (0.644)	0.956 (0.247)	1.179 (0.306)	1.587 (0.638)	1.139 (0.315)	1.499 (0.384)	1.486 (0.433)
Henan	0.790 (0.202)	0.588^*^ (0.175)	1.392 (0.359)	1.005 (0.259)	1.154 (0.299)	1.791 (0.720)	1.268 (0.350)	1.569^*^ (0.401)	1.666^*^ (0.4845)
Hubei	0.890 (0.227)	0.622 (0.185)	1.934^**^ (0.500)	1.072 (0.277)	1.205 (0.313)	2.277^**^ (0.916)	1.493 (0.413)	1.738^**^ (0.445)	1.930^**^ (0.562)
Hunan	1.070 (0.273)	0.731 (0.217)	2.129^***^ (0.550)	1.246 (0.322)	1.505 (0.391)	2.730^**^ (1.097)	1.774^**^ (0.490)	2.158^***^ (0.552)	2.286^***^ (0.665)
Guangdong	0.960 (0.246)	0.541^**^ (0.161)	1.927^**^ (0.500)	0.983 (0.255)	1.150 (0.300)	2.108^*^ (0.849)	1.241 (0.344)	1.732^**^ (0.445)	1.976^**^ (0.577)
Guangxi	0.945 (0.242)	0.457^***^ (0.136)	1.795^**^ (0.465)	1.002 (0.259)	0.899 (0.234)	2.404^**^ (0.967)	1.504 (0.416)	2.666^***^ (0.684)	2.282^***^ (0.665)
Hainan	0.480^***^ (0.125)	0.386^***^ (0.116)	1.503 (0.395)	0.603^*^ (0.158)	0.742 (0.196)	1.401 (0.570)	1.067 (0.299)	1.882^**^ (0.489)	1.460 (0.430)
Chongqing	1.039 (0.266)	0.690 (0.206)	1.765^**^ (0.457)	1.393 (0.360)	1.364 (0.355)	2.911^***^ (1.171)	2.130^***^ (0.589)	2.032^***^ (0.521)	2.064^**^ (0.601)
Sichuan	0.860 (0.219)	0.541 ^**^ (0.161)	1.467 (0.378)	1.111 (0.287)	1.017 (0.264)	2.091^*^ (0.840)	1.766^**^ (0.487)	1.935^**^ (0.495)	1.992^**^ (0.579)
Guizhou	0.716 (0.183)	0.428^***^ (0.127)	1.313 (0.340)	0.966 (0.250)	1.163 (0.303)	1.843 (0.742)	1.813^**^ (0.502)	1.827^**^ (0.469)	1.949^**^ (0.568)
Yunan	0.656 (0.168)	0.433^***^ (0.129)	1.587^*^ (0.412)	0.948 (0.246)	1.010 (0.263)	1.723 (0.695)	1.614^*^ (0.448)	2.661^***^ (0.685)	1.830^**^ (0.535)
Tibet	0.183 ^***^ (0.050)	0.188^***^ (0.057)	0.502^**^ (0.133)	0.629^*^ (0.168)	0.432^***^ (0.116)	1.178 (0.486)	2.016^**^ (0.571)	1.754^**^ (0.462)	1.629 (0.486)
Shannxi	0.670 (0.172)	0.557 ^**^ (0.166)	1.144 (0.296)	1.013 (0.262)	1.110 (0.289)	1.530 (0.617)	1.314 (0.364)	1.111 (0.285)	1.507 (0.440)
Gansu	0.655^*^ (0.168)	0.492^**^ (0.147)	1.451 (0.376)	1.000 (0.259)	0.996 (0.259)	1.712 (0.689)	1.579^*^ (0.437)	1.613^*^ (0.414)	1.753^*^ (0.511)
Qinghai	0.721 (0.187)	0.496^**^ (0.149)	1.197 (0.314)	1.126 (0.295)	1.362 (0.359)	1.770 (0.719)	2.321^***^ (0.649)	1.734^**^ (0.451)	2.155^***^ (0.634)
Ningxia	0.720 (0.187)	0.588^*^ (0.177)	1.583^*^ (0.416)	1.210 (0.317)	1.000 (0.264)	1.724 (0.701)	1.658^*^ (0.464)	1.421 (0.369)	2.024^**^ (0.596)
Xinjiang	0.580^**^ (0.151)	0.446 ^***^ (0.134)	2.099^***^ (0.552)	1.260 (0.330)	1.044 (0.275)	1.646 (0.669)	2.442^***^ (0.683)	4.993^***^ (1.309)	2.152^***^ (0.633)
XPCC	1.193 (0.400)	1.021 (0.400)	1.444 (0.500)	2.140^**^ (0.729)	1.155 (0.397)	1.571 (0.781)	2.057^**^ (0.723)	3.036^***^ (1.067)	2.375^**^ (0.864)
Beijing	0.879 (0.224)	3.133^***^ (0.929)	1.385 (0.356)	0.722 (0.186)	1.48 (0.383)	0.127^***^ (0.051)	0.442^***^ (0.122)	0.824 (0.210)	0.348^***^ (0.101)
Pseudo *R*^2^	0.0077	0.0065	0.0123	0.0065	0.0057	0.0091	0.0146	0.0168	0.0086

*Standard deviations in parentheses*.

***, **, and

* *represent 1,5, and 10%*.

### Association Between Geodemographic Factors and Types of Access to Health Knowledge

Regarding types of access to health knowledge, after orthogonal rotation method for Caesar normalization after three iterations, PCA showed 2 factors (eigenvalues >1.0) were L1 (eigenvalue = 3.535) and L2 (eigenvalue = 1.023) had commutative contribution rate 50.65%. Cronbach's alphas of the two factors (L1: kntypea, kntyped, kntypef, kntypeg, kntypei; L2: kntypeb, kntypec, kntypee, kntypeh) were 0.7750 and 0.6159, respectively. This showed the factor analysis was highly acceptable.

**Figure 5:**
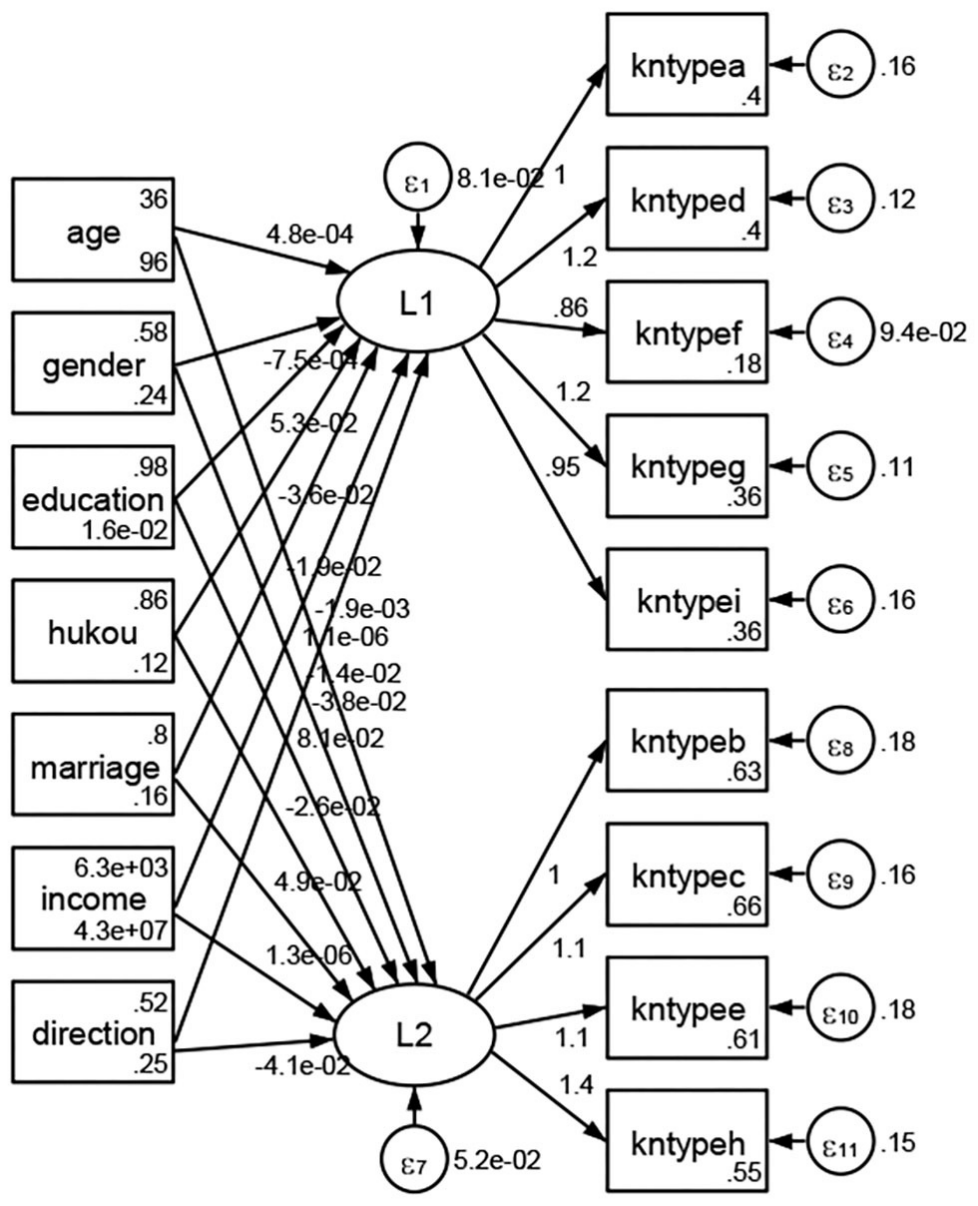
Total sample.

**Figure 6:**
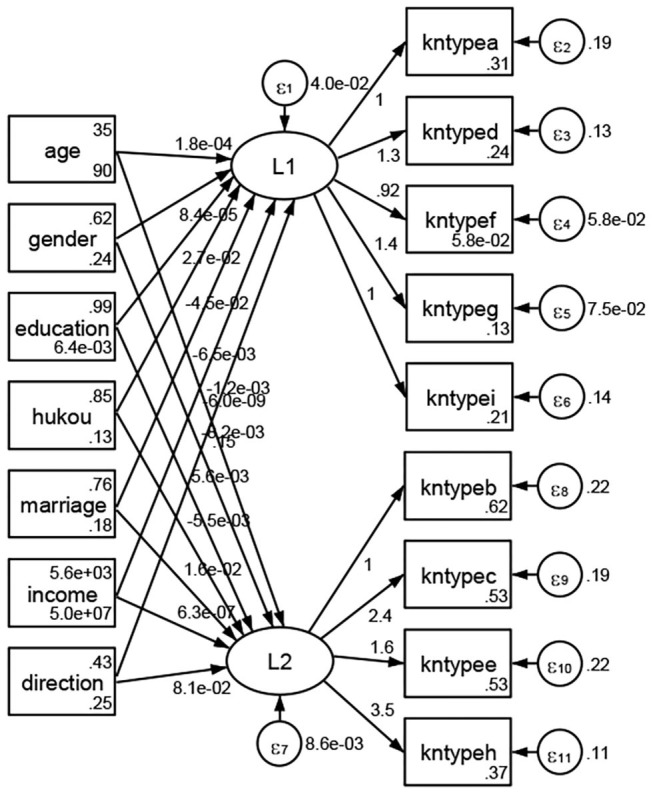
Hebei.

**Figure 7:**
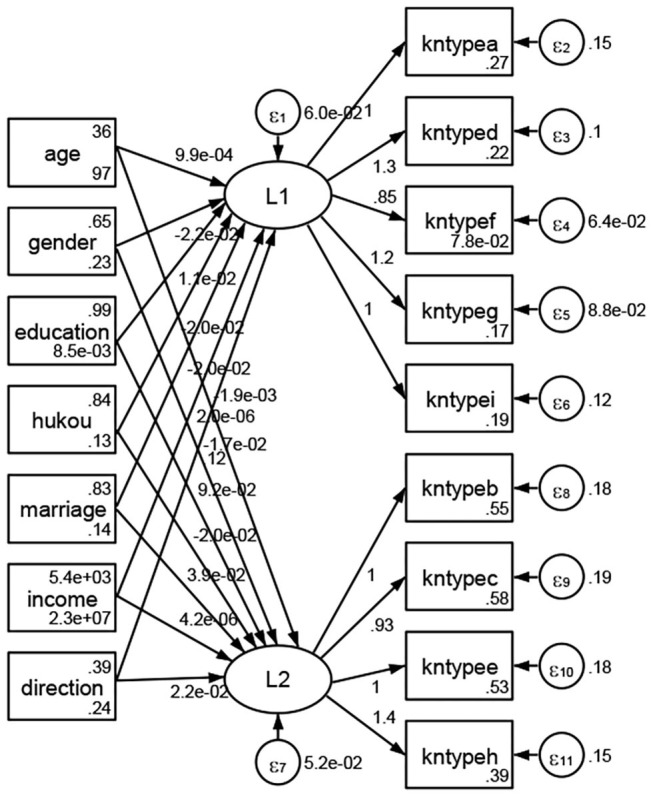
Shanxi.

**Figure 8:**
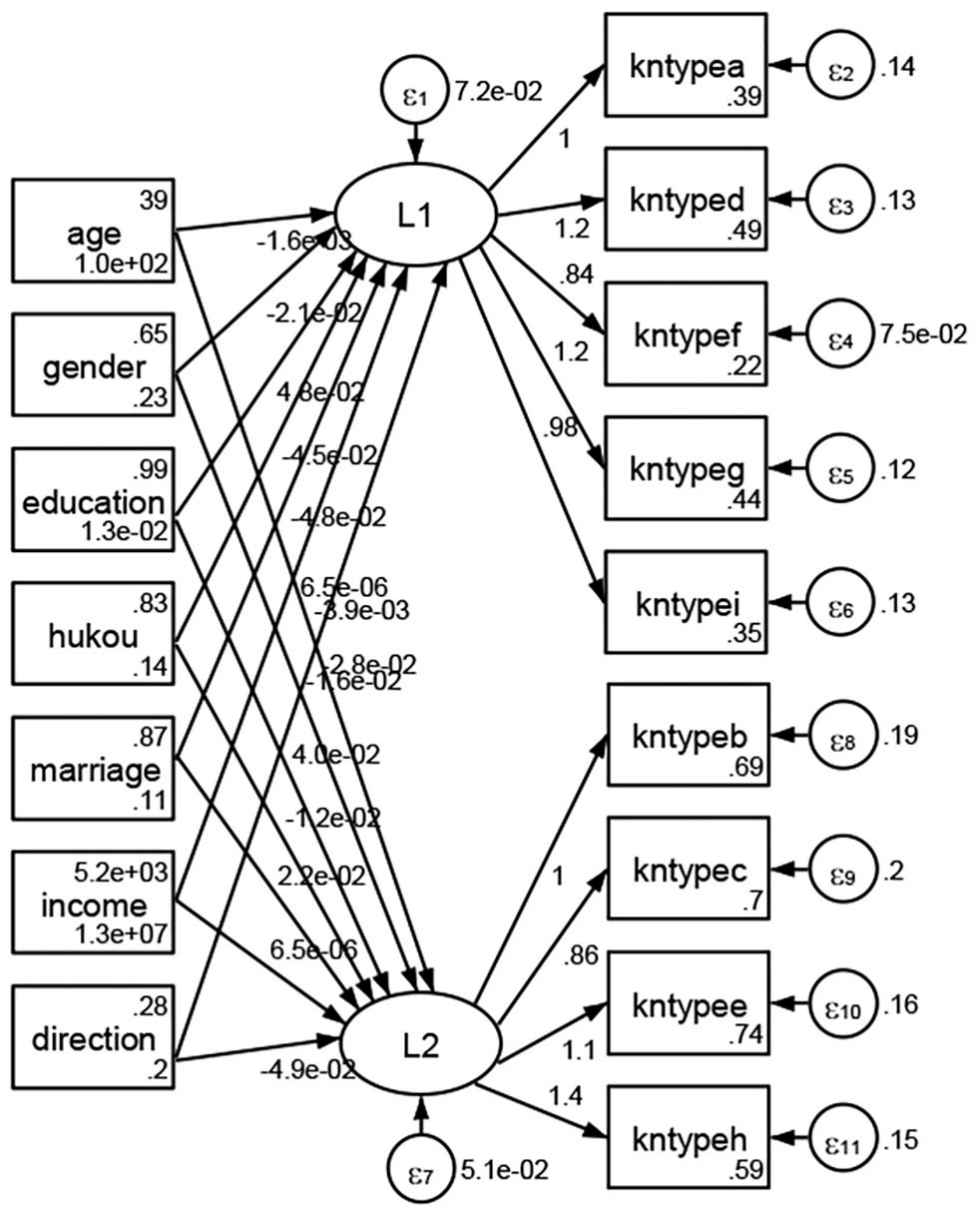
Inner Mongolia.

**Figure 9:**
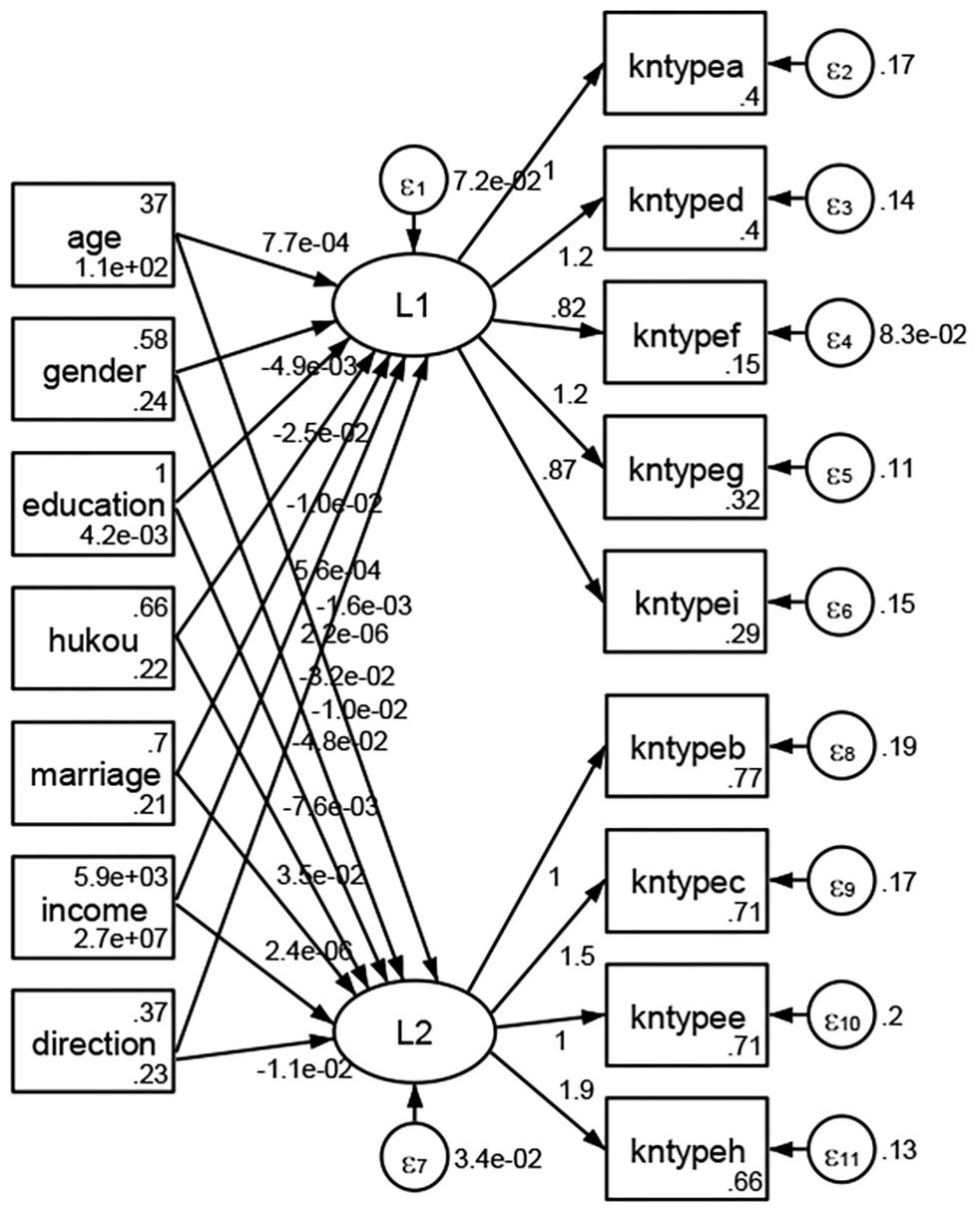
Liaoning.

**Figure 10:**
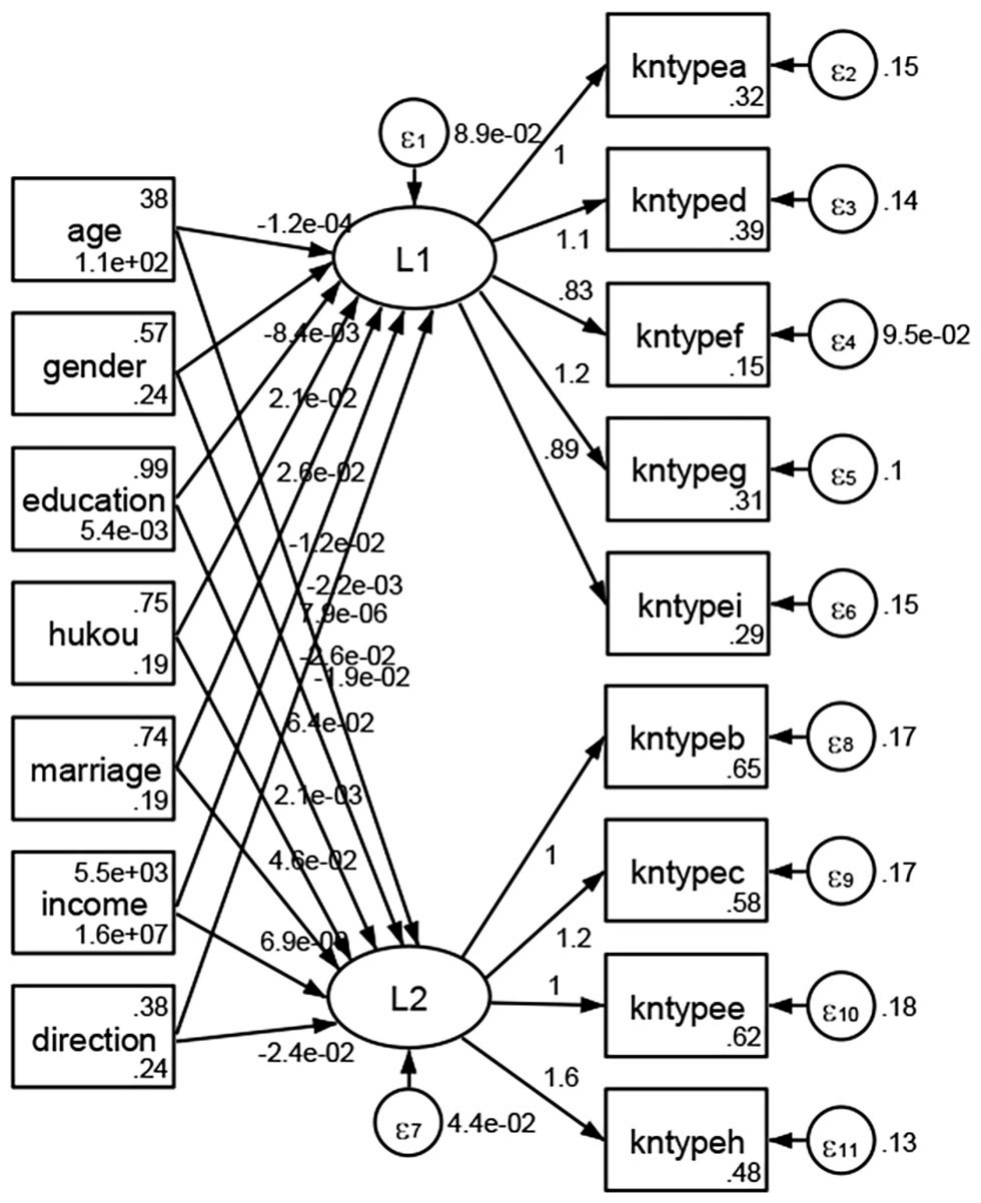
Jilin.

**Figure 11:**
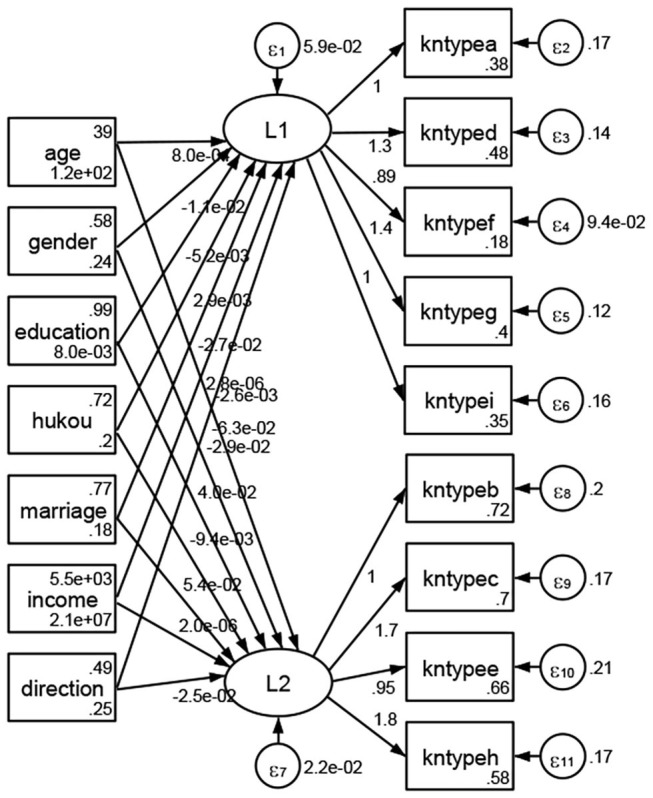
Heilongjiang.

**Figure 12:**
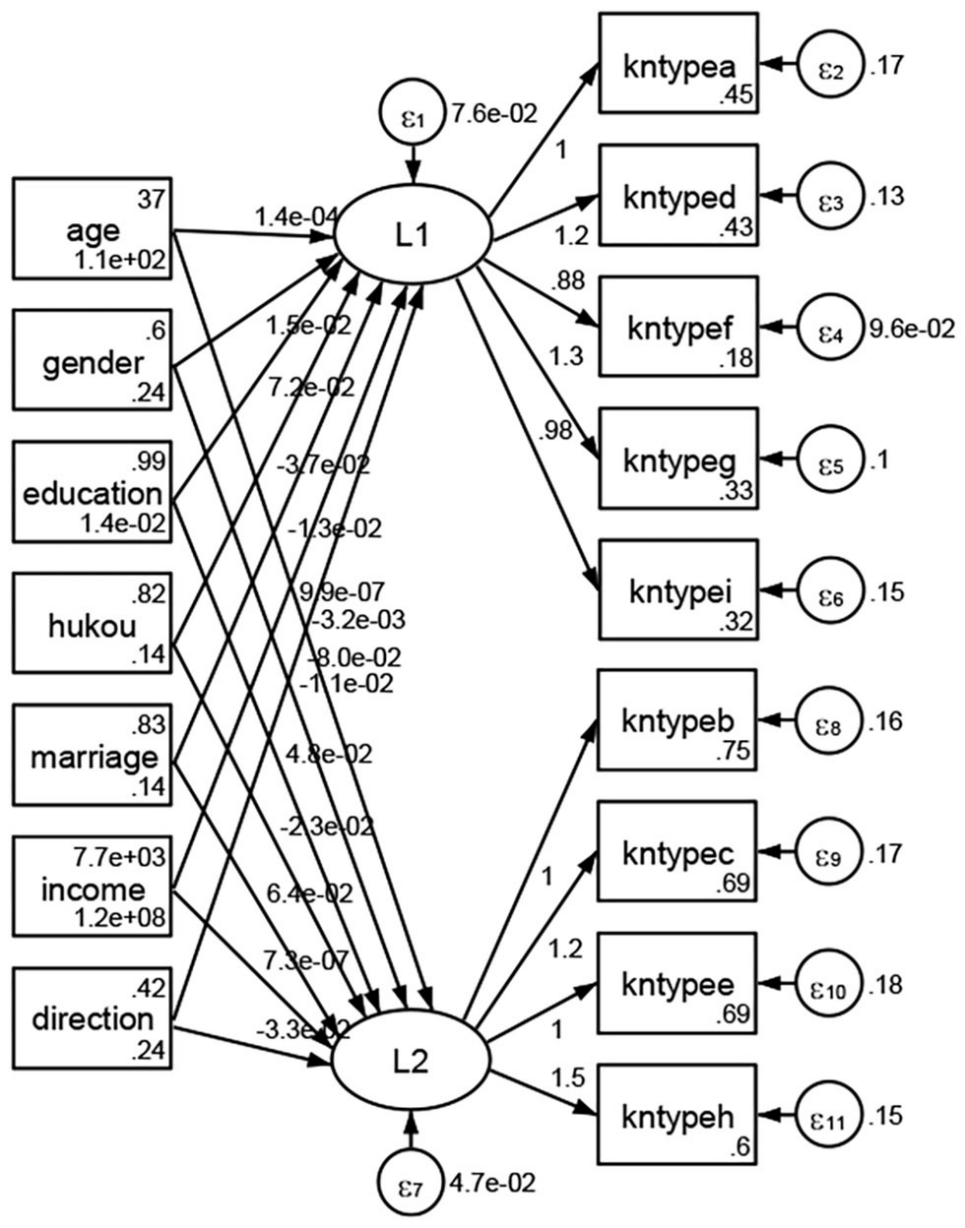
Jiangsu.

**Figure 13:**
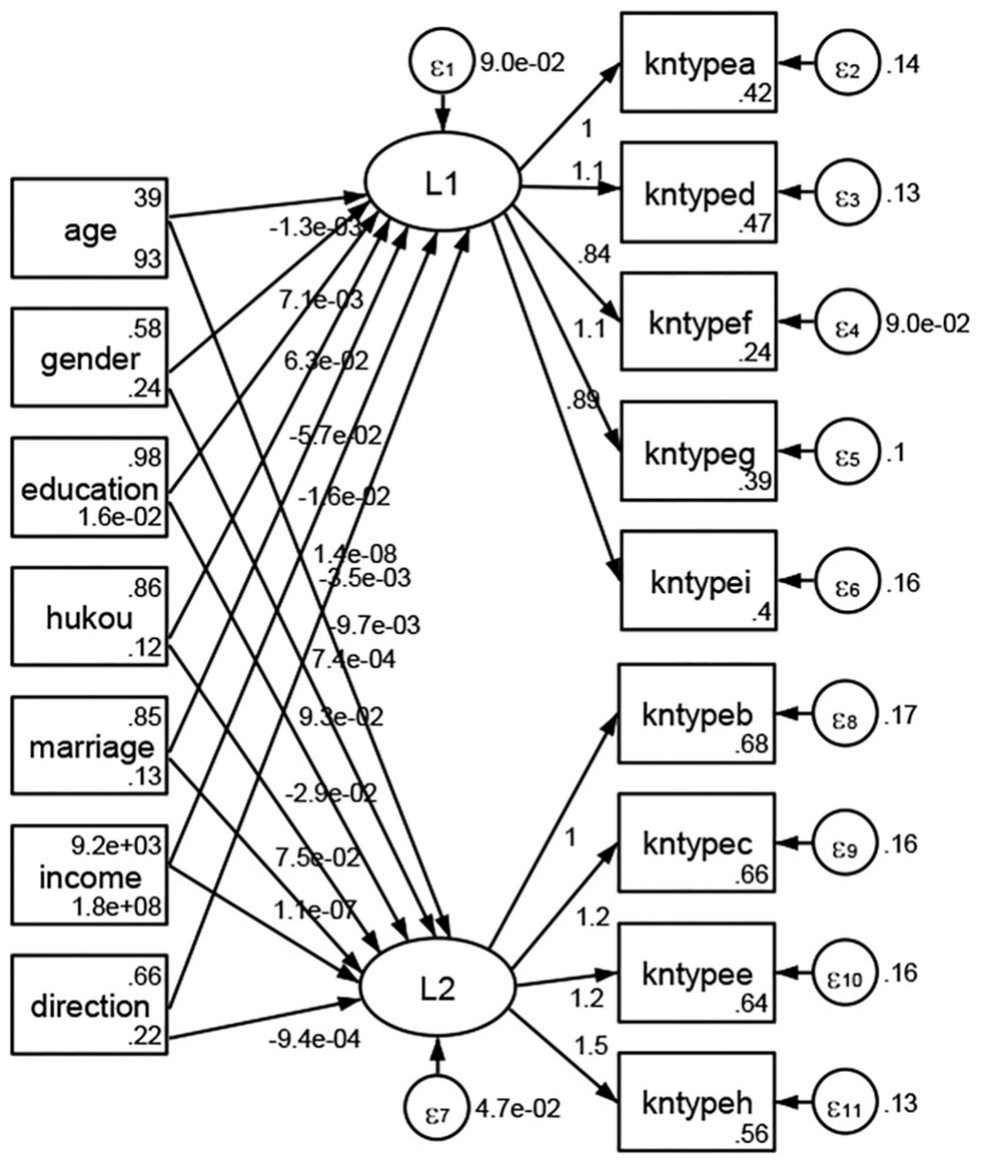
Zhejiang.

**Figure 14:**
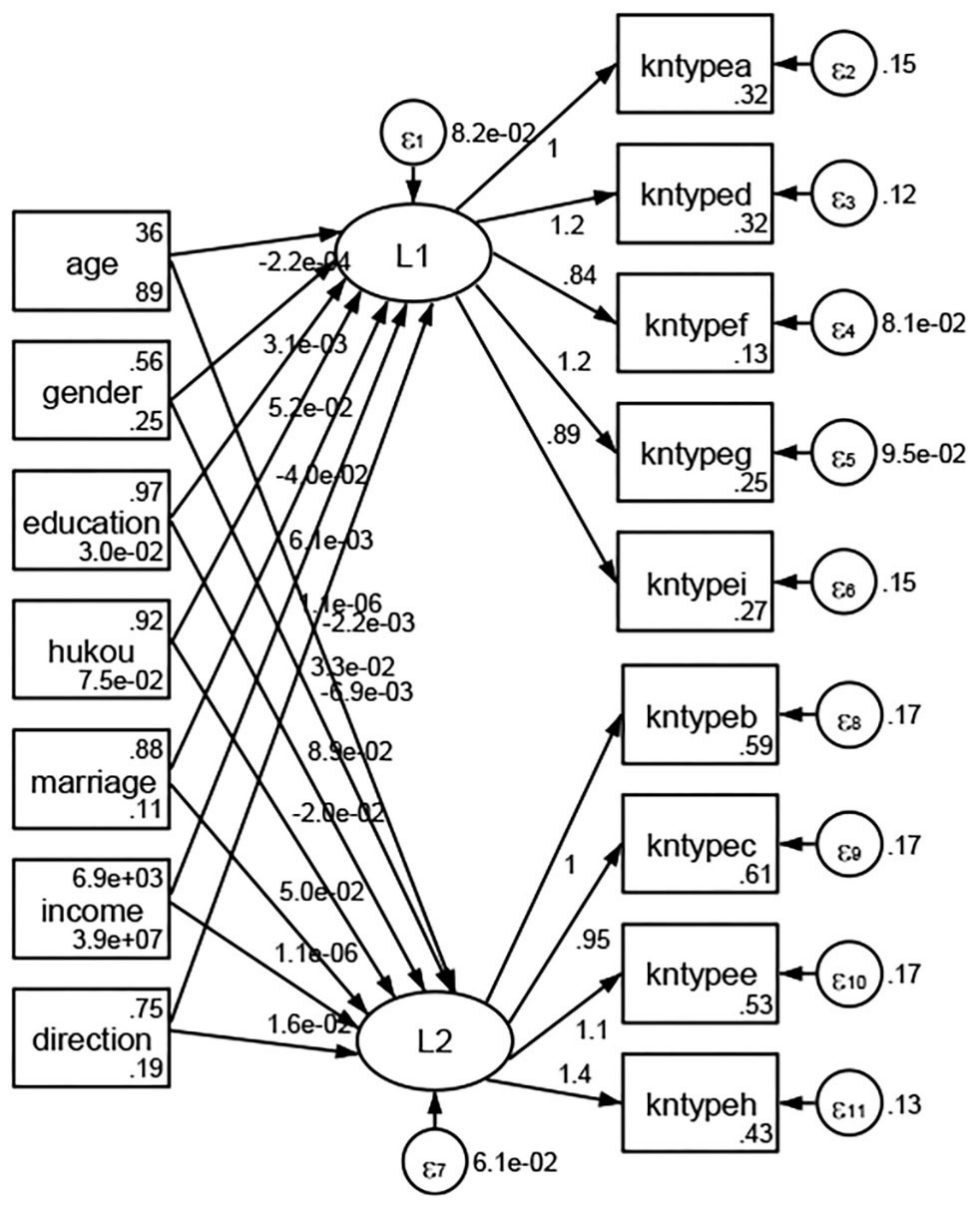
Anhui.

**Figure 15:**
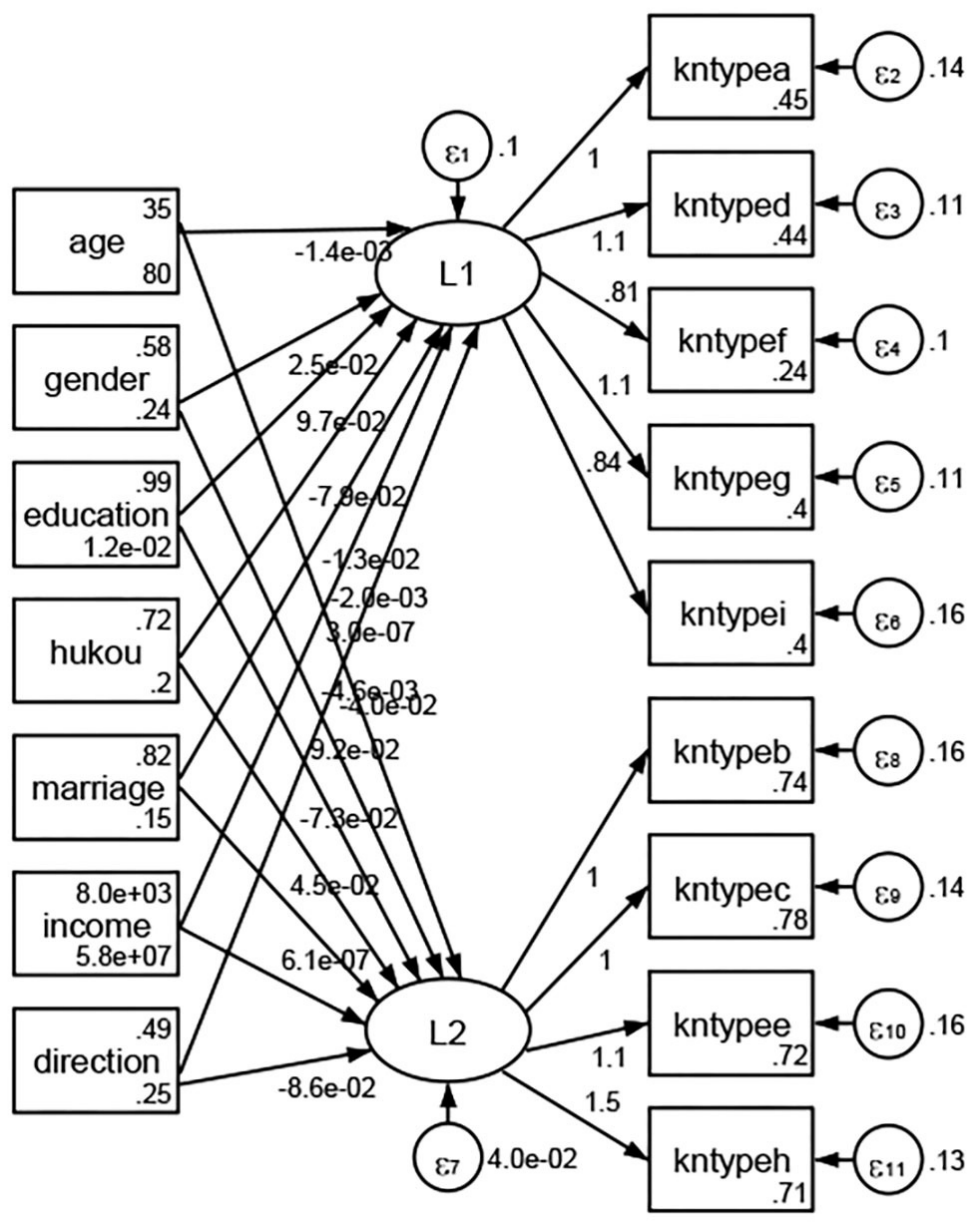
Fujian.

**Figure 16:**
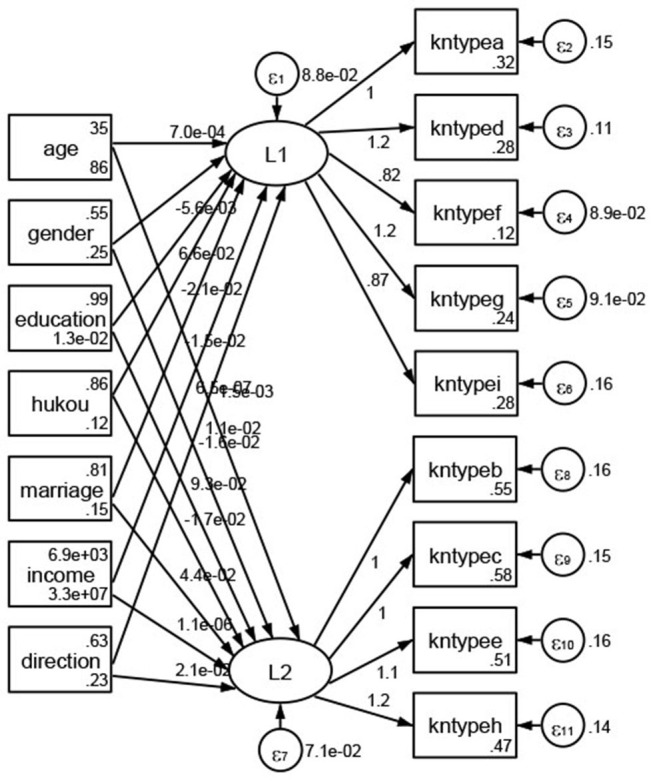
Jiangxi.

**Figure 17:**
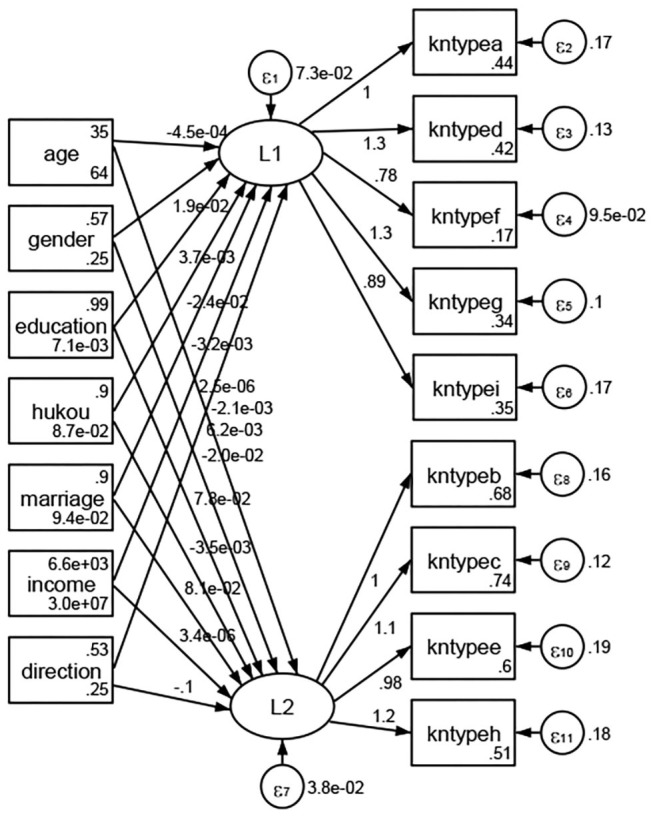
Shandong.

**Figure 18:**
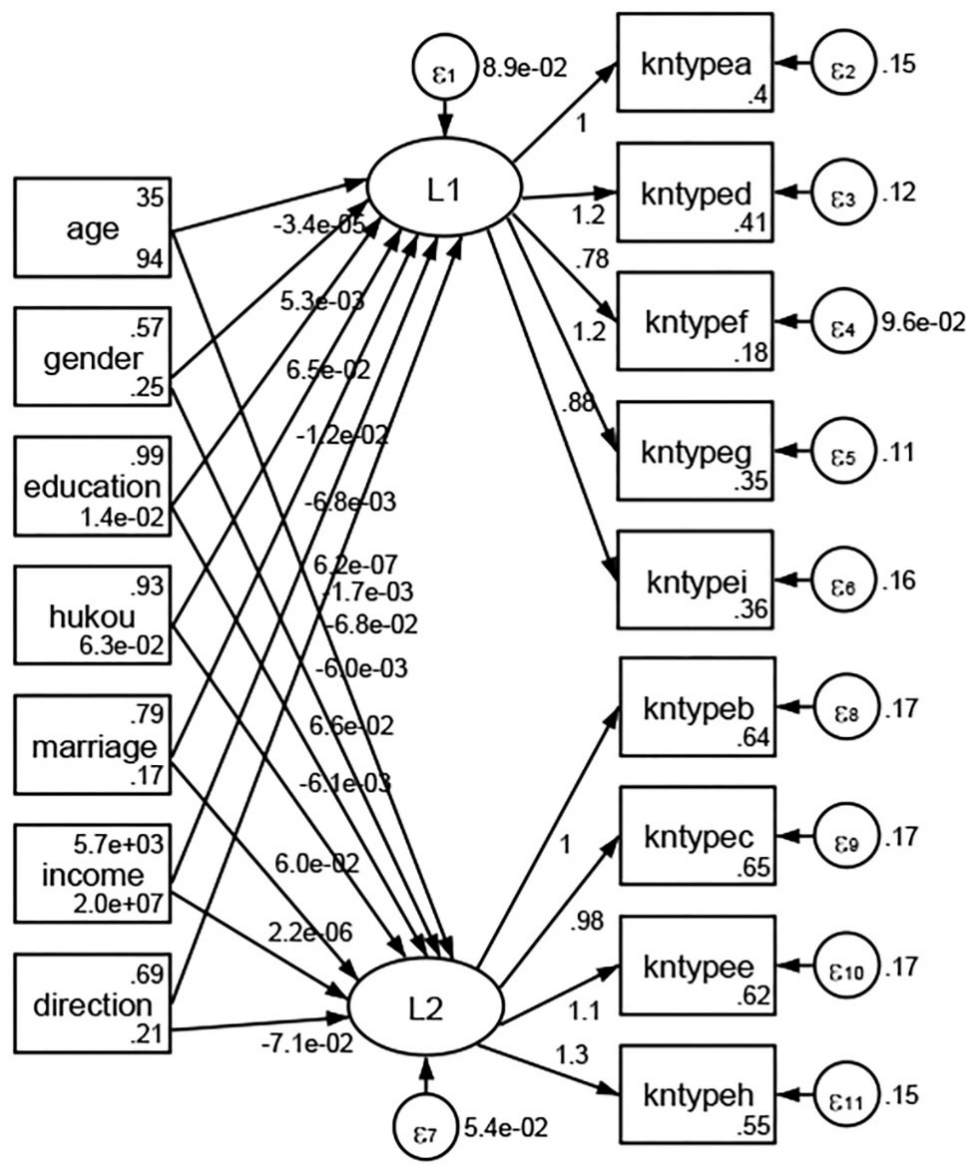
Henan.

**Figure 19:**
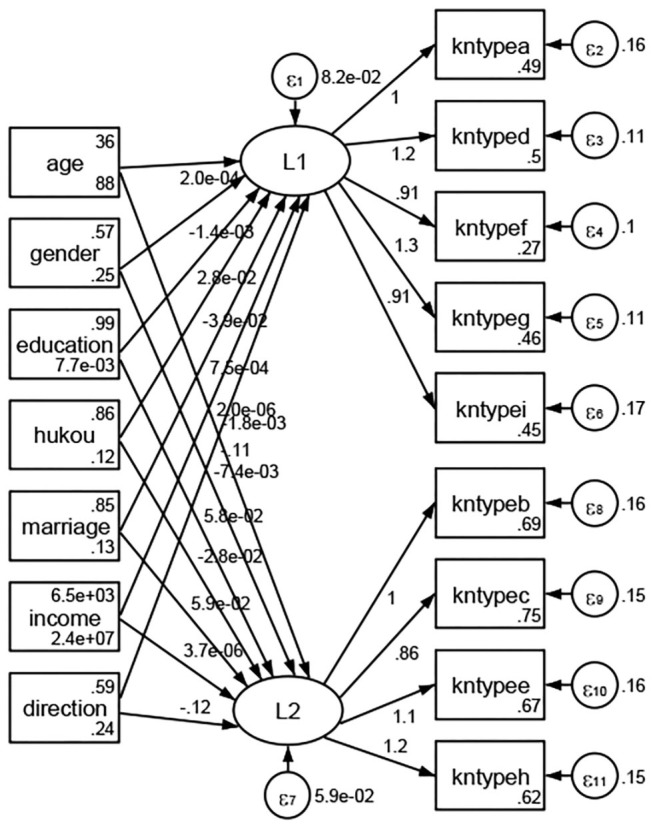
Hubei.

**Figure 20:**
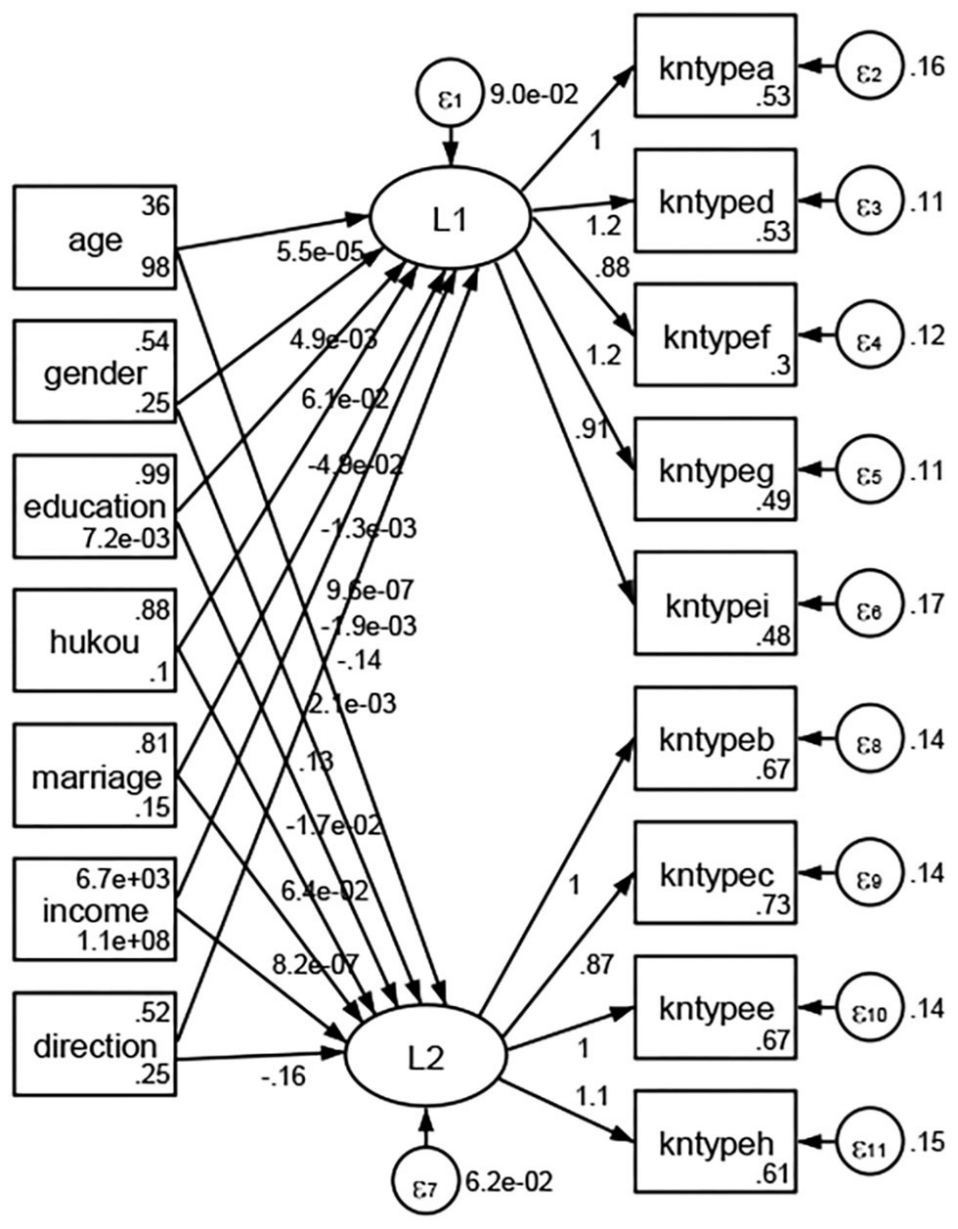
Hunan.

**Figure 21:**
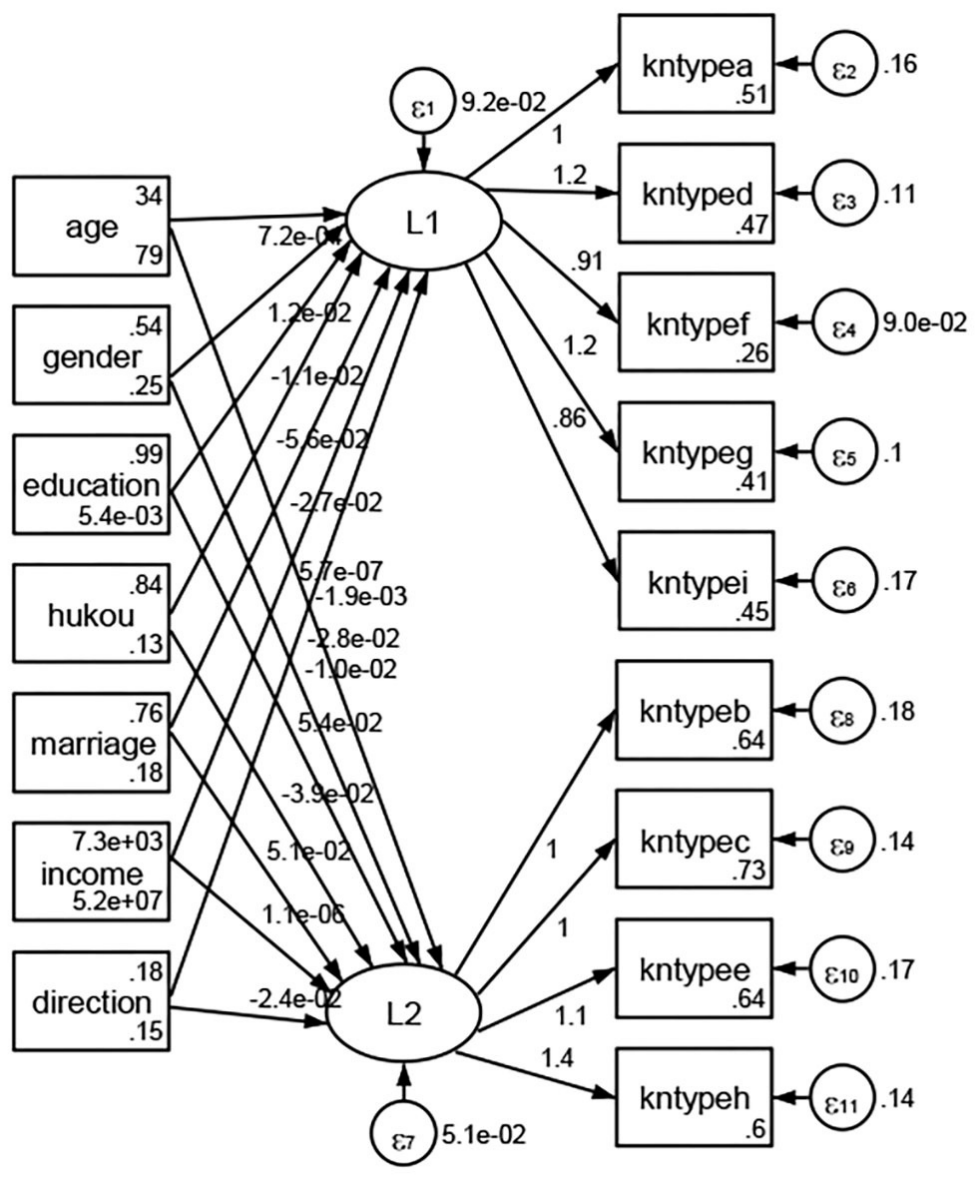
Guangdong.

**Figure 22:**
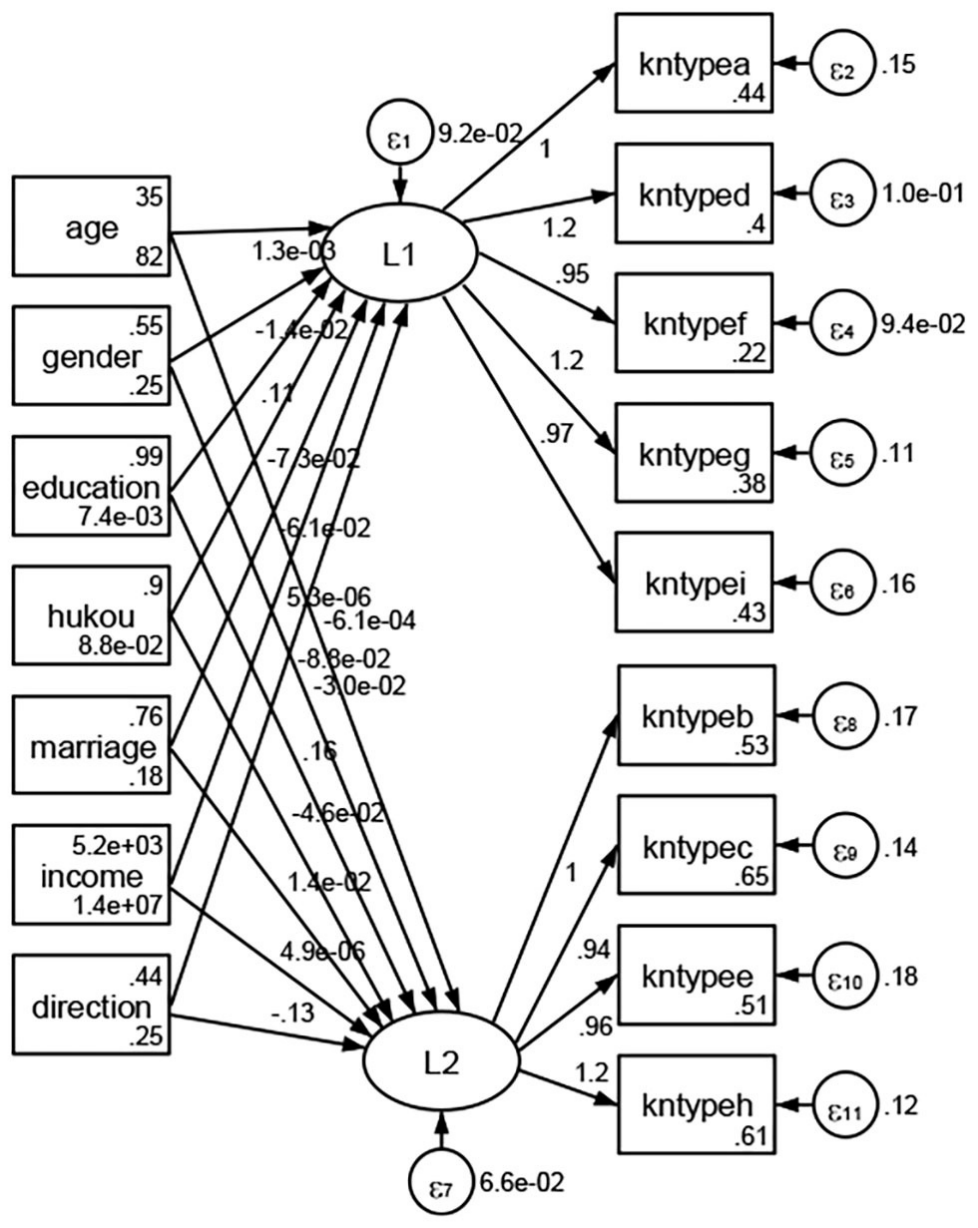
Guangxi.

**Figure 23:**
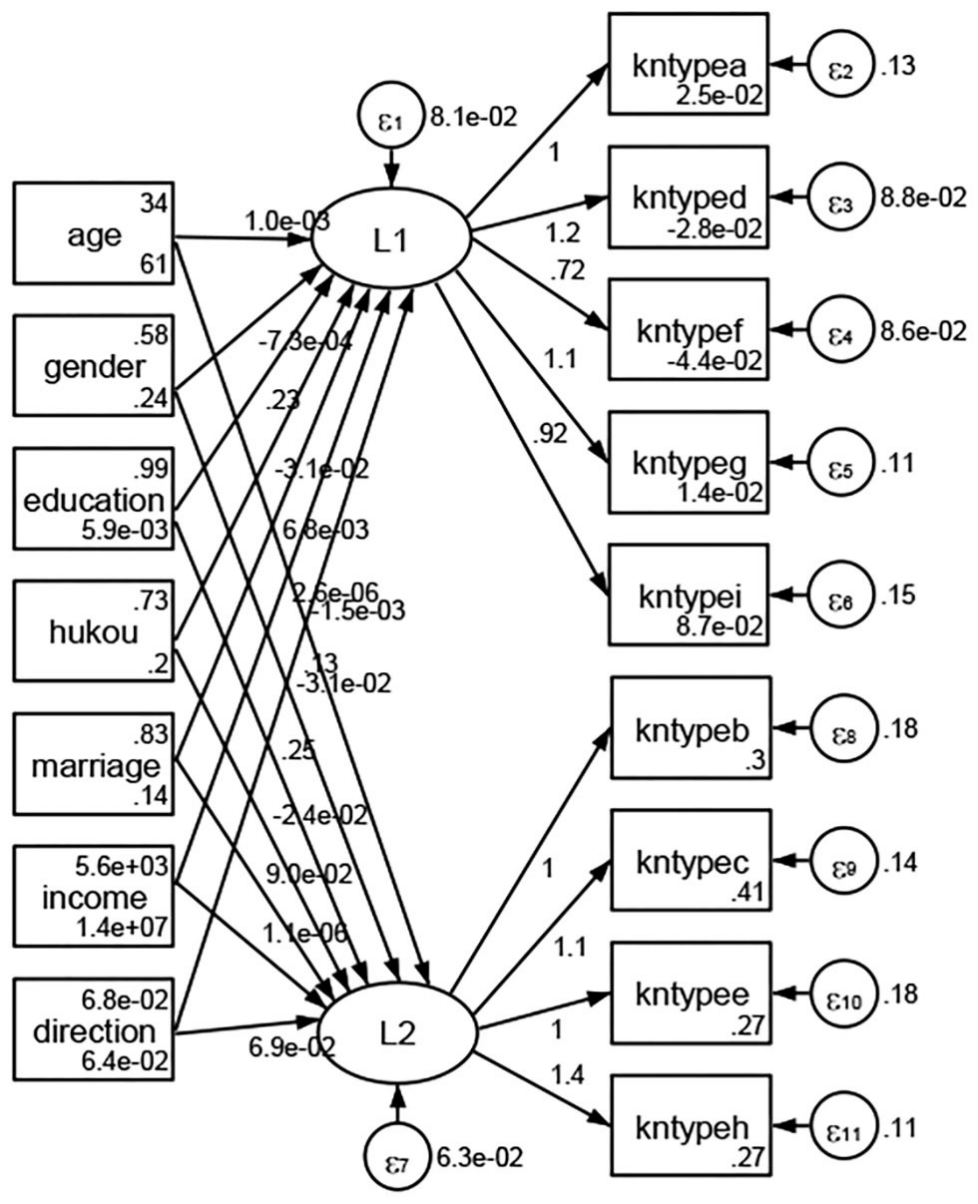
Hainan.

**Figure 24:**
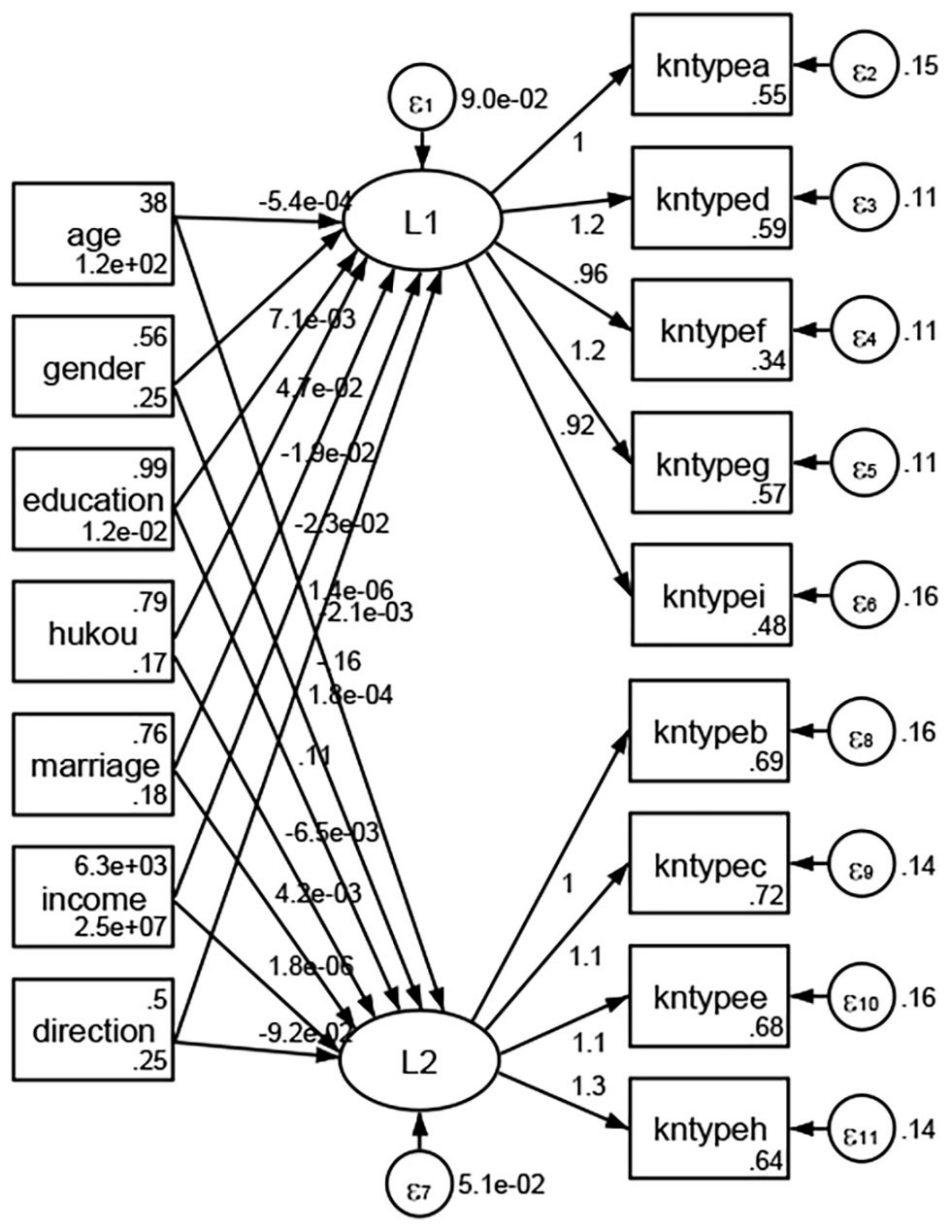
Chongqing.

**Figure 25:**
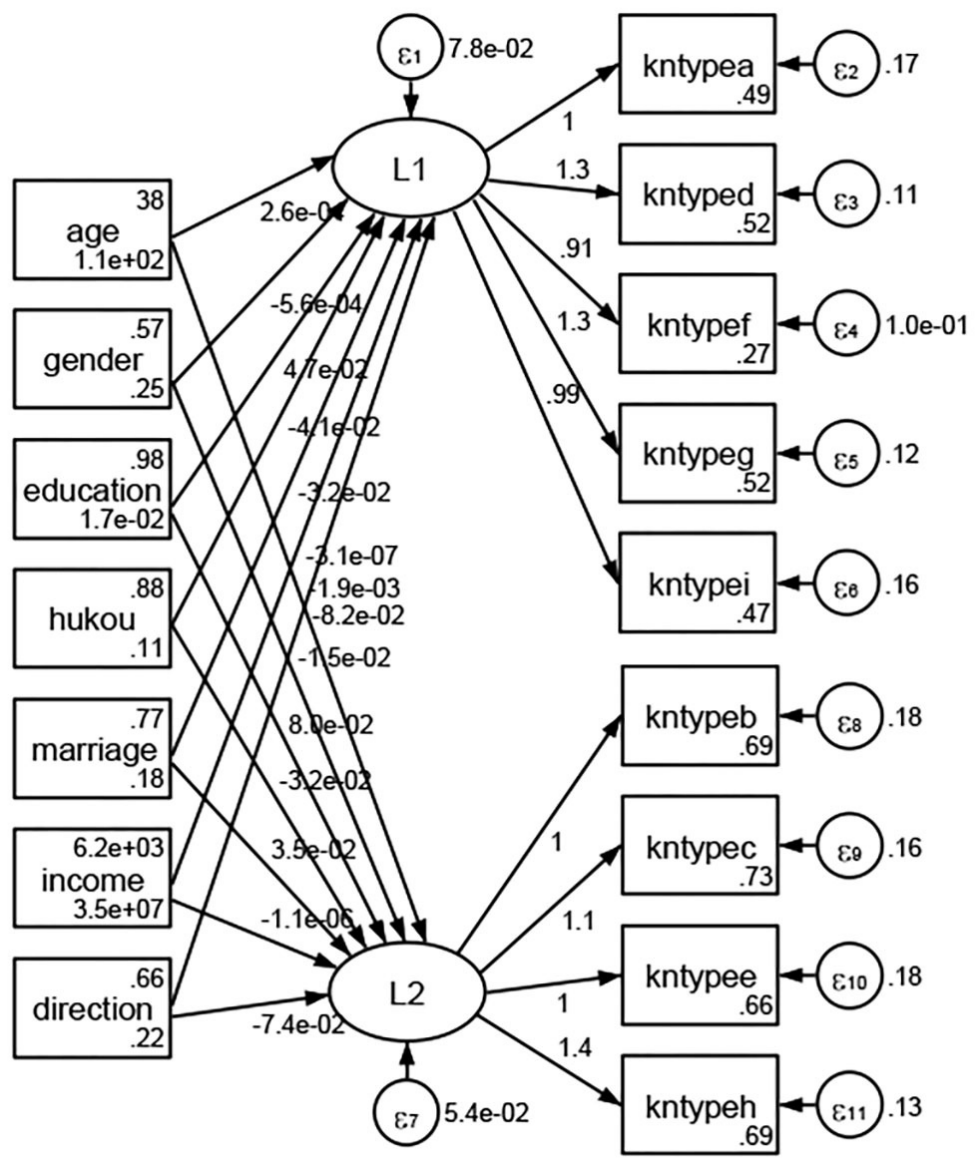
Sichuan.

See Figures 5–34. Except Beijing, Tianjin, and Shanghai, RMSEA values in total sample (RMSEA = 0.086), Hebei (RMSEA = 0.087), Shanxi (RMSEA = 0.081), Inner Mongolia (RMSEA = 0.090), Liaoning (RMSEA = 0.087), Jilin (RMSEA = 0.088), Heilongjiang (RMSEA = 0.082), Jiangsu (RMSEA = 0.089), Zhejiang (RMSEA = 0.083), Anhui (RMSEA = 0.089), Fujian (RMSEA = 0.085), Jiangxi (RMSEA = 0.084), Shandong (RMSEA = 0.083), Henan (RMSEA = 0.086), Hubei (RMSEA = 0.086), Hunan (RMSEA = 0.085), Guangdong (RMSEA = 0.090), Guangxi (RMSEA = 0.094), Hainan (RMSEA = 0.096), Chongqing (RMSEA = 0.091), Sichuan (RMSEA = 0.088), Guizhou (RMSEA = 0.096), Yunan (RMSEA = 0.097), Tibet (RMSEA = 0.096), Shannxi (RMSEA = 0.089), Gansu (RMSEA = 0.087), Qinghai (RMSEA = 0.091), Ningxia (RMSEA = 0.095), Xinjiang (RMSEA = 0.090), and XPCC (RMSEA =0.086) were <0.1. Thus, provincial differences were reflected by the associations between geodemographic factors and types of access to health knowledge.

**Figure 26:**
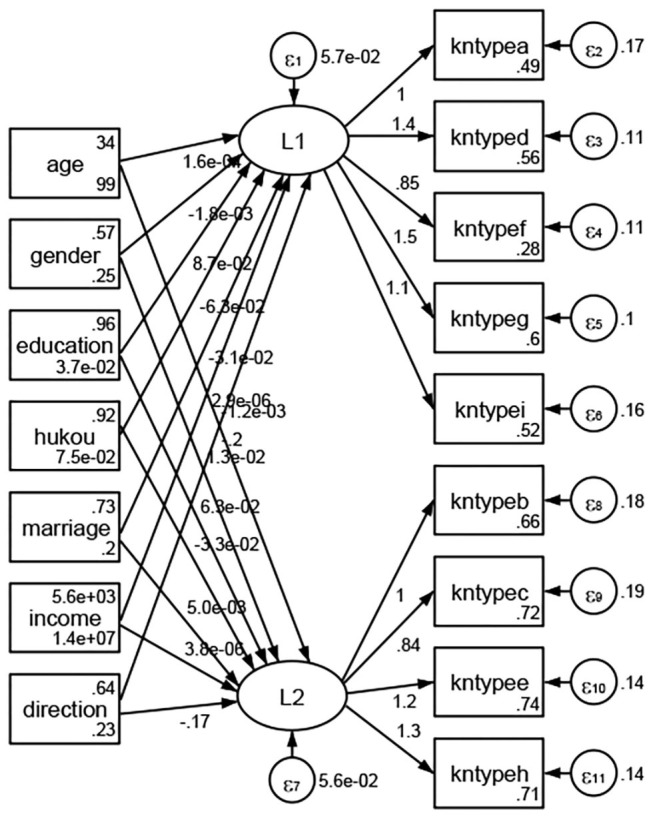
Guizhou.

**Figure 27:**
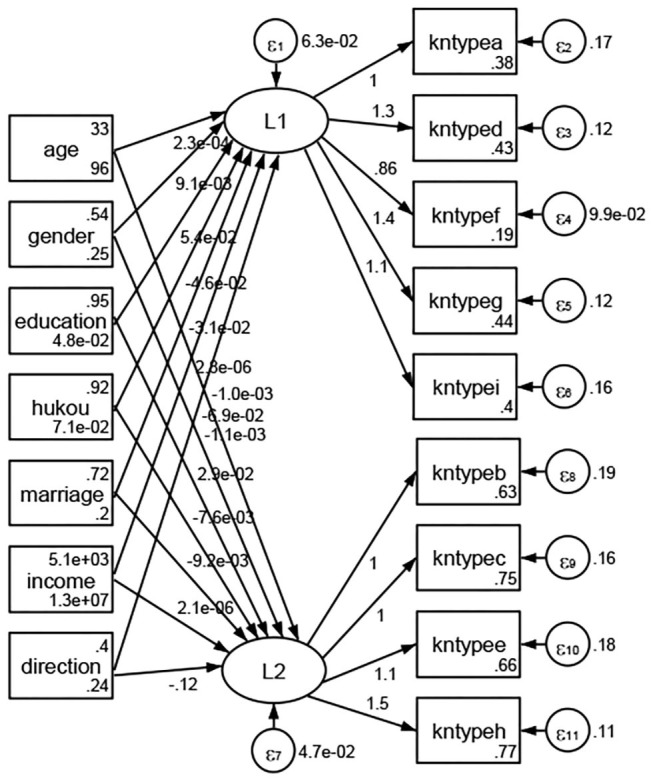
Yunan.

**Figure 28:**
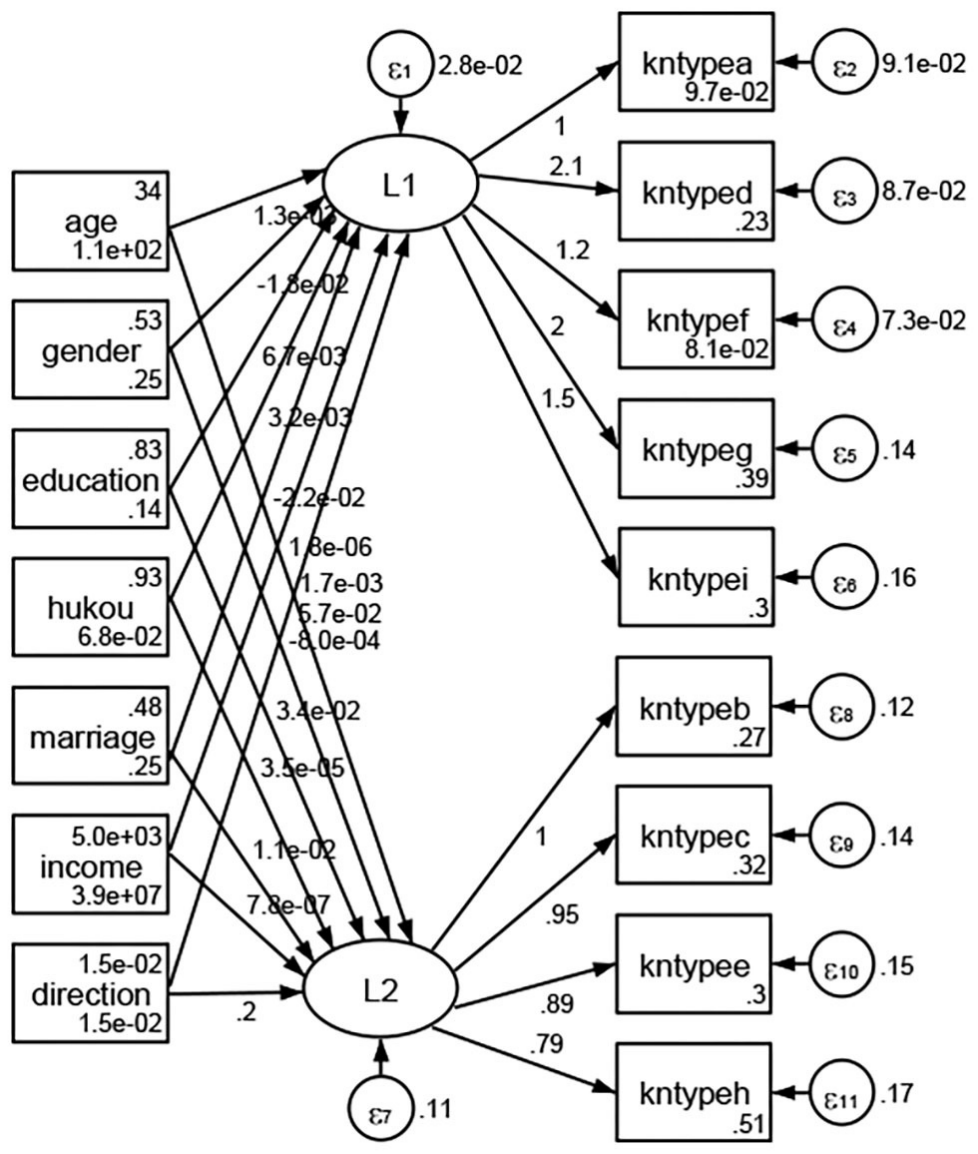
Tibet.

**Figure 29:**
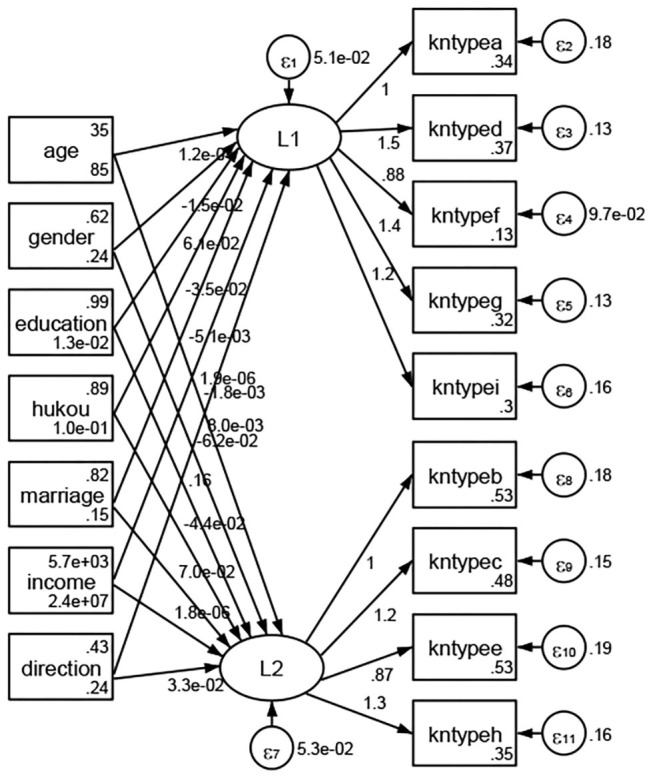
Shannxi.

**Figure 30:**
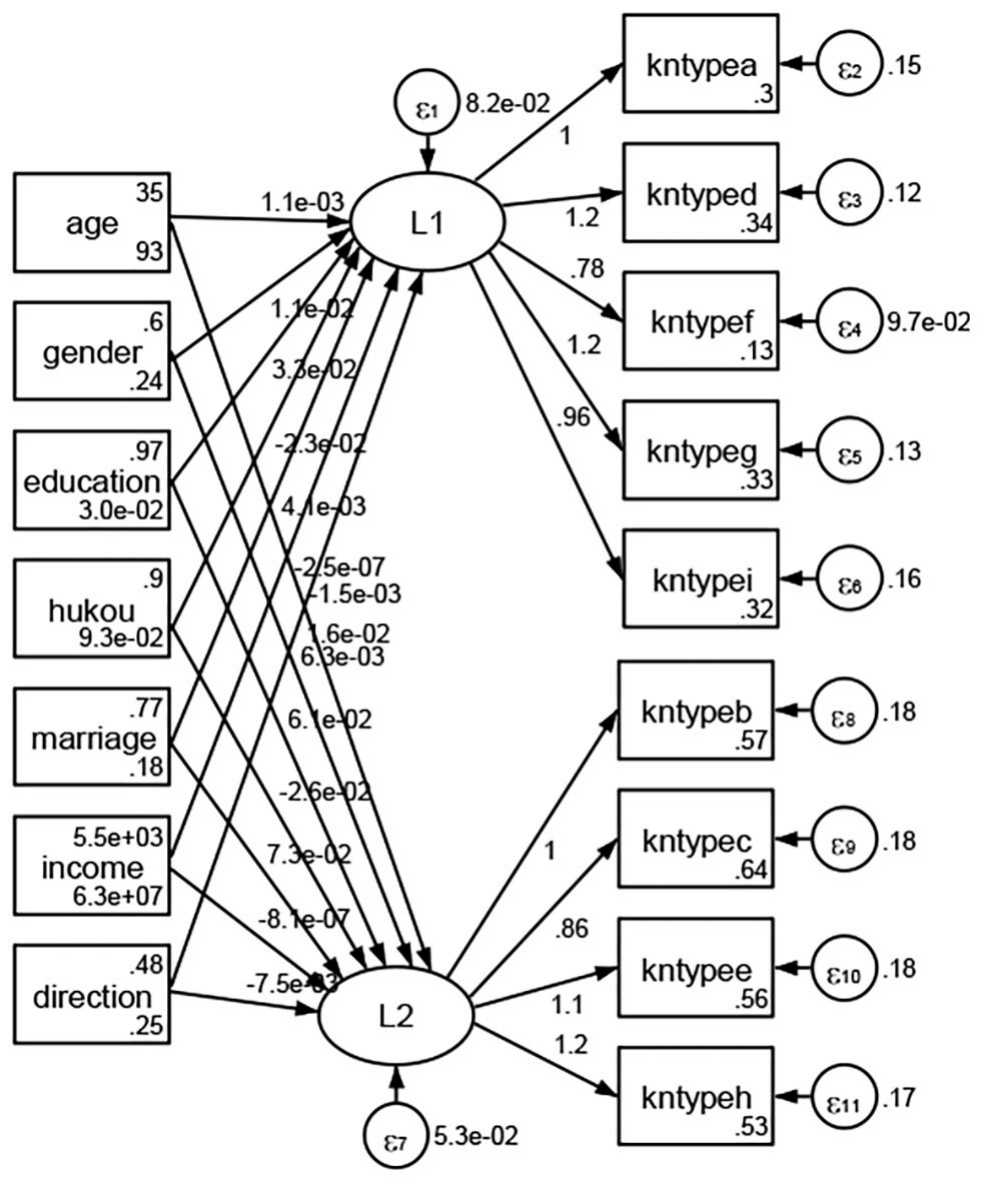
Gansu.

**Figure 31:**
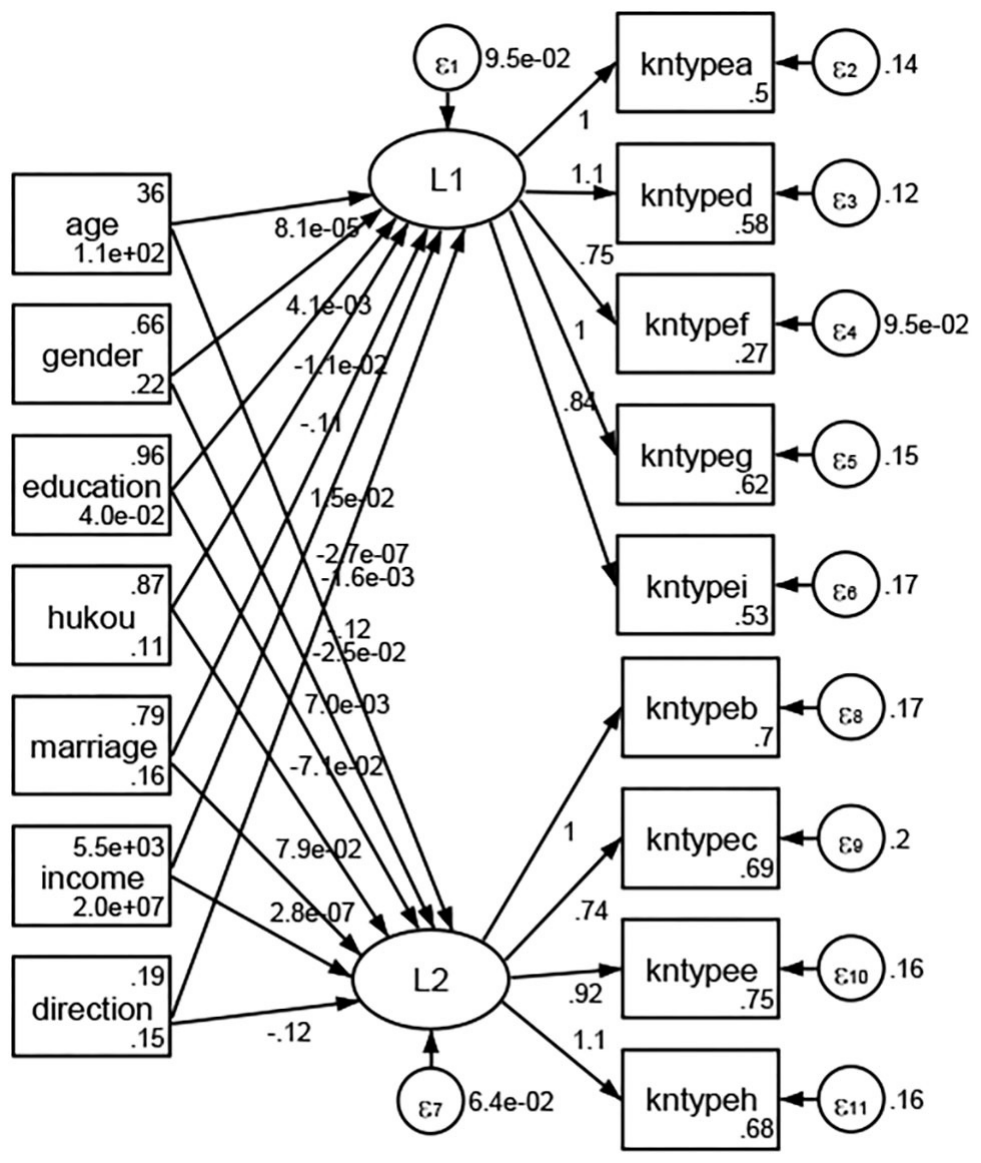
Qinghai.

**Figure 32:**
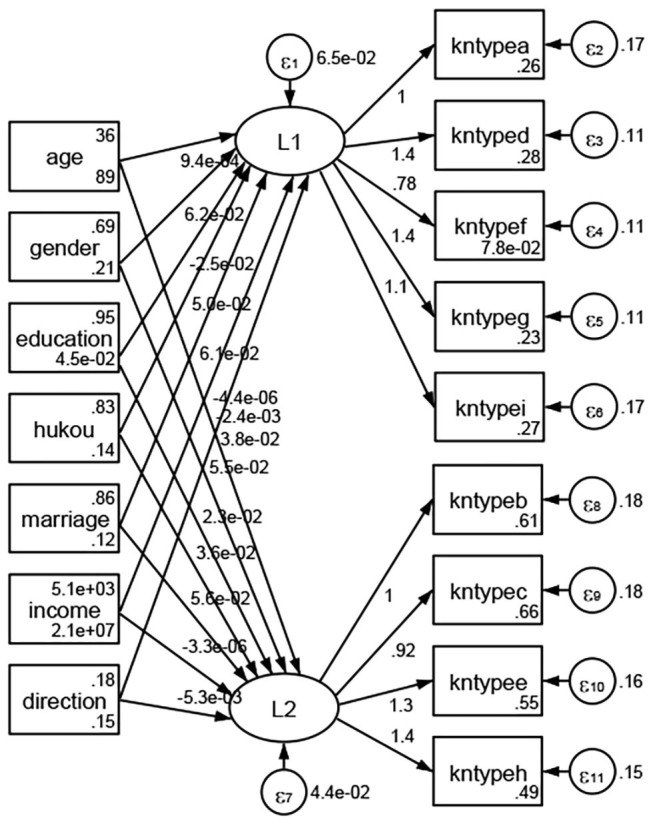
Ningxia.

**Figure 33:**
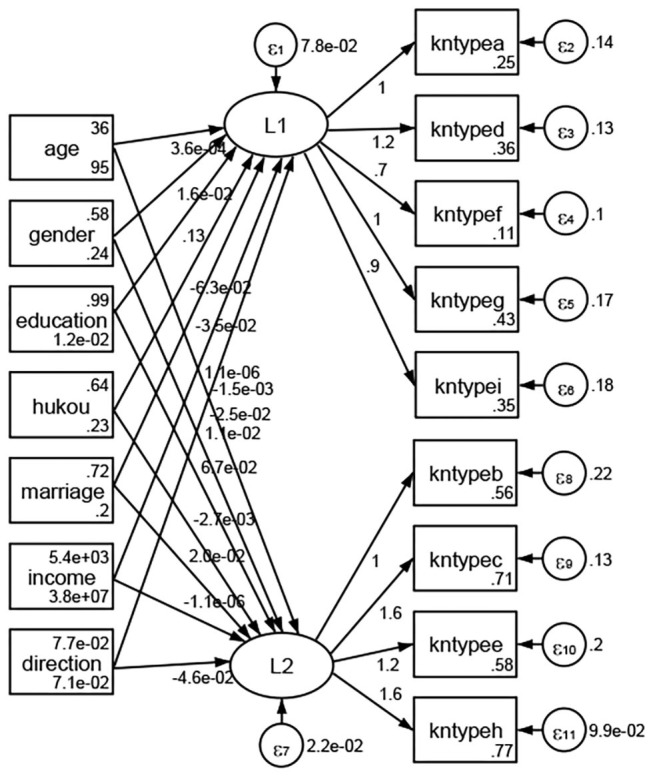
Xinjiang.

**Figure 34:**
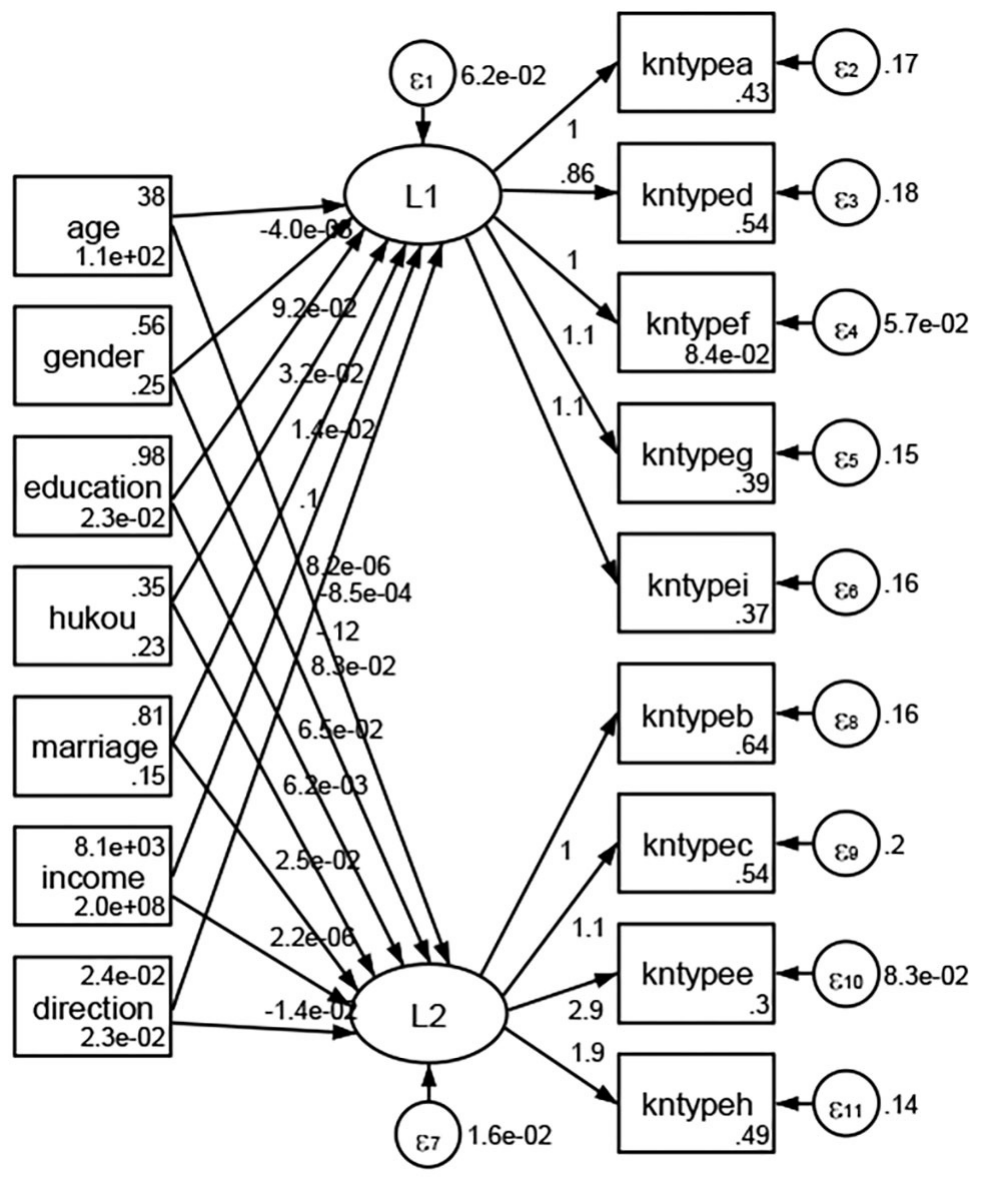
XPCC.

### Association Between Geodemographic Factors and Methods of Access to Health Knowledge

Regarding methods of access to health knowledge, after using the orthogonal rotation method for Caesar normalization for three iterations, PCA showed two factors (eigenvalues >1.0) were L1 (eigenvalue = 2.012) and L2 (eigenvalue = 1.516) had a commutative contribution rate of 44.10%. Cronbach's alphas of the two factors (L1: knpathb, knpathc, knpathe, knpathf, knpathg, knpathh; L2: knpatha, knpathd) were 0.5015 and 0.5499, respectively. This showed the factor analysis was moderately acceptable.

**Figure 35:**
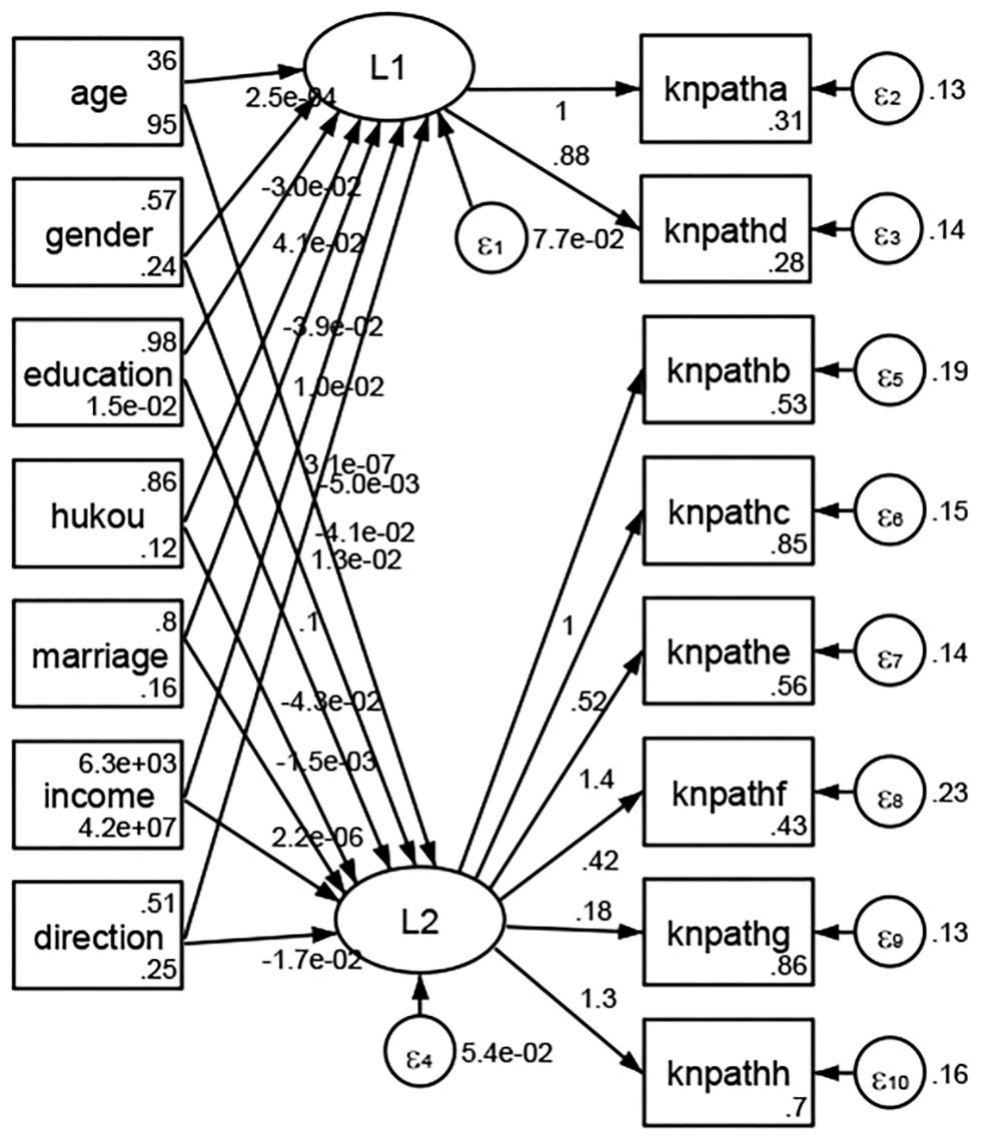
Total sample.

**Figure 36:**
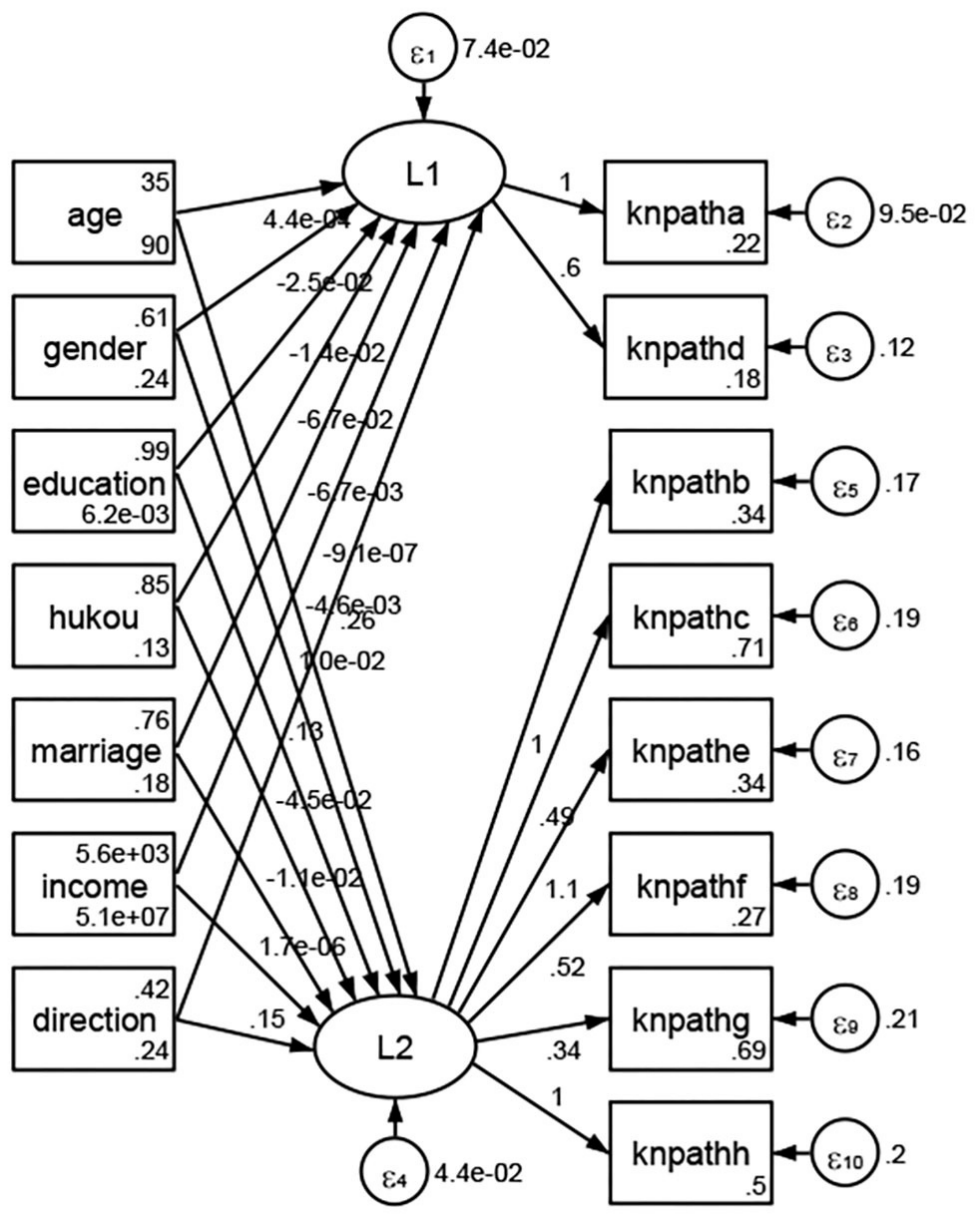
Hebei.

**Figure 37:**
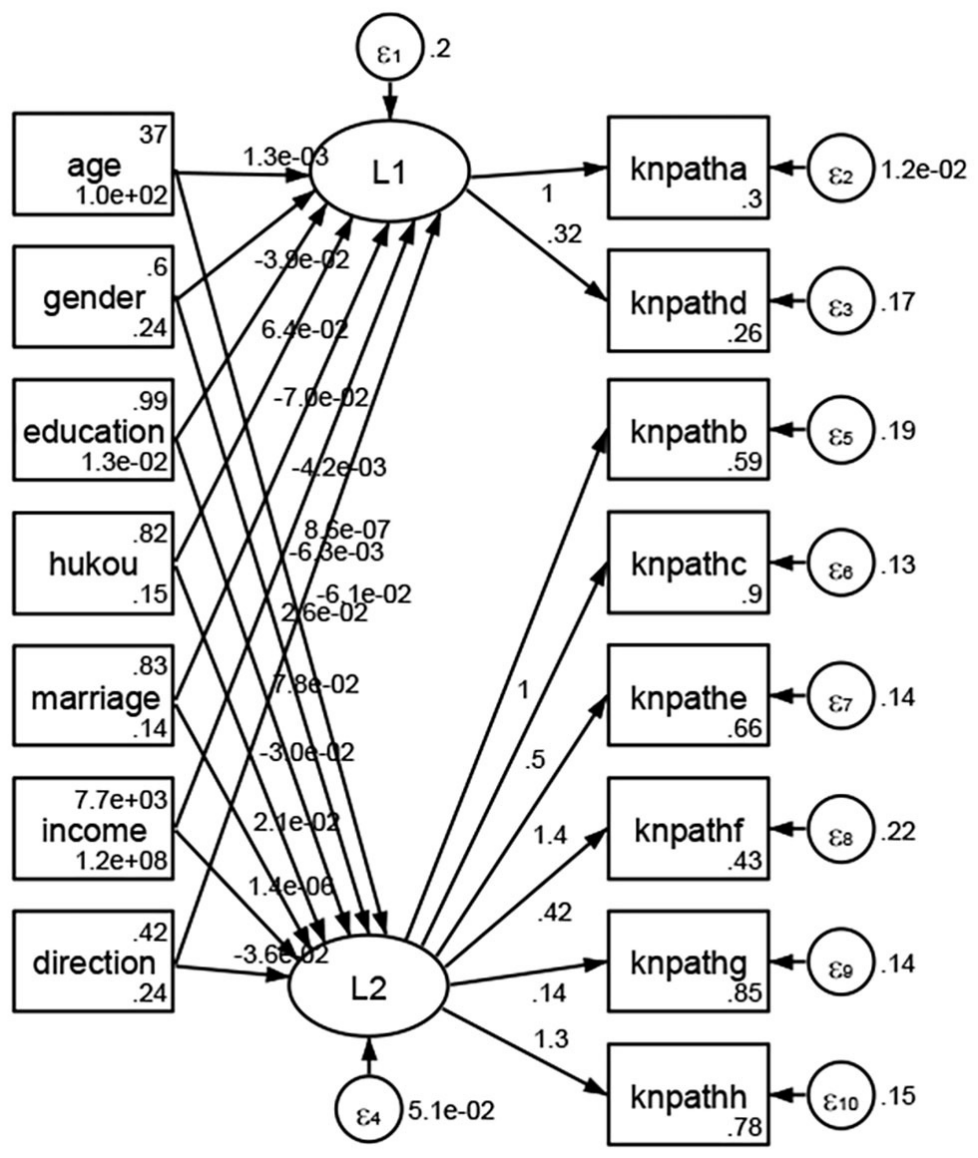
Jiangsu.

**Figure 38:**
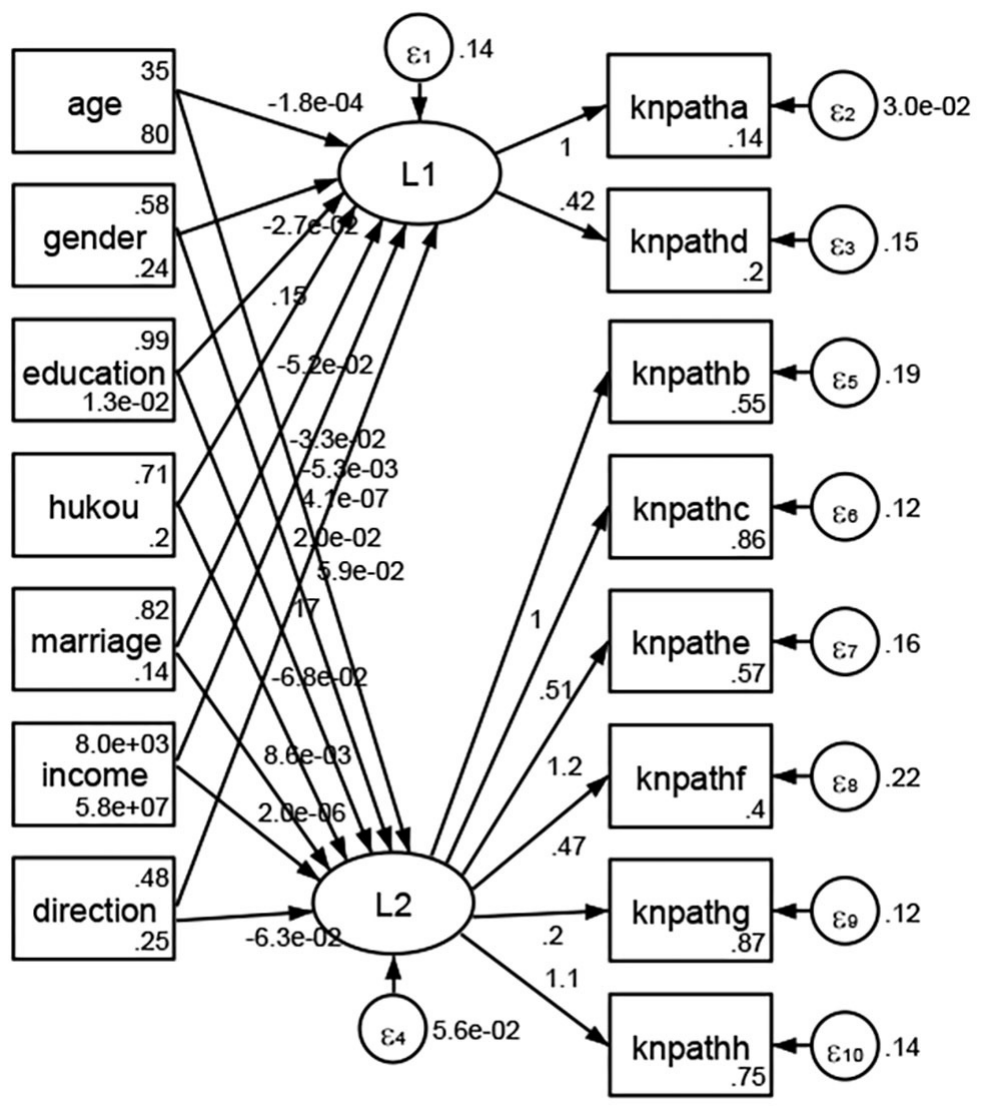
Fujian.

**Figure 39:**
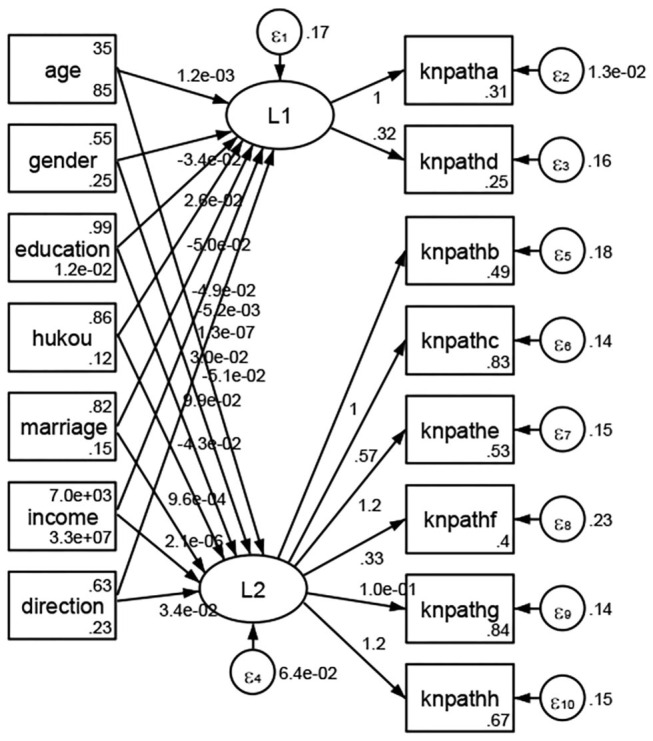
Jiangxi.

**Figure 40:**
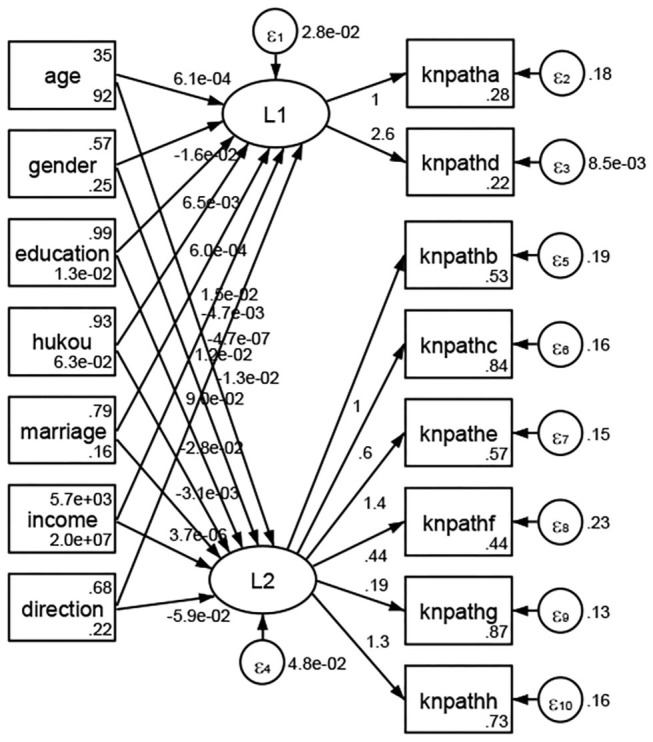
Henan.

**Figure 41:**
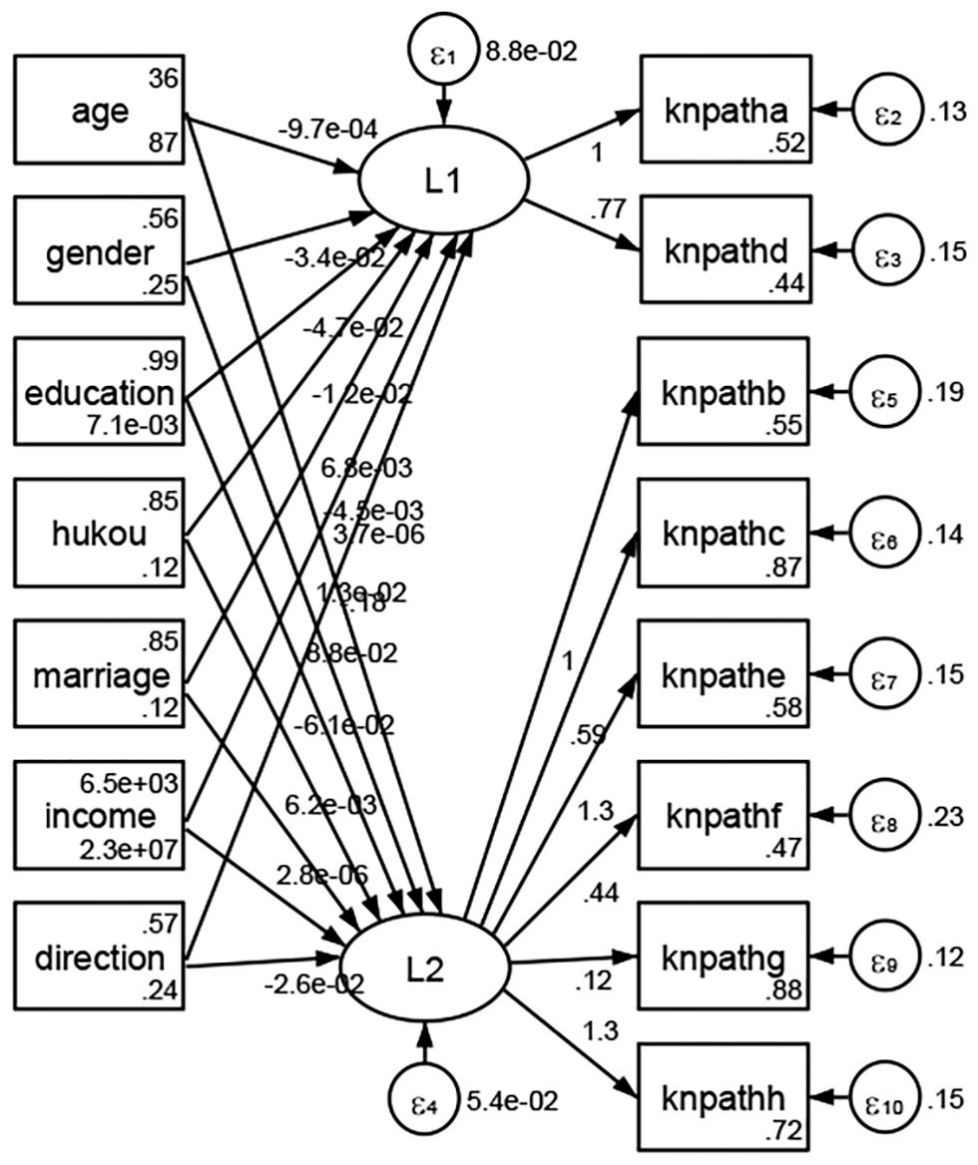
Hubei.

**Figure 42:**
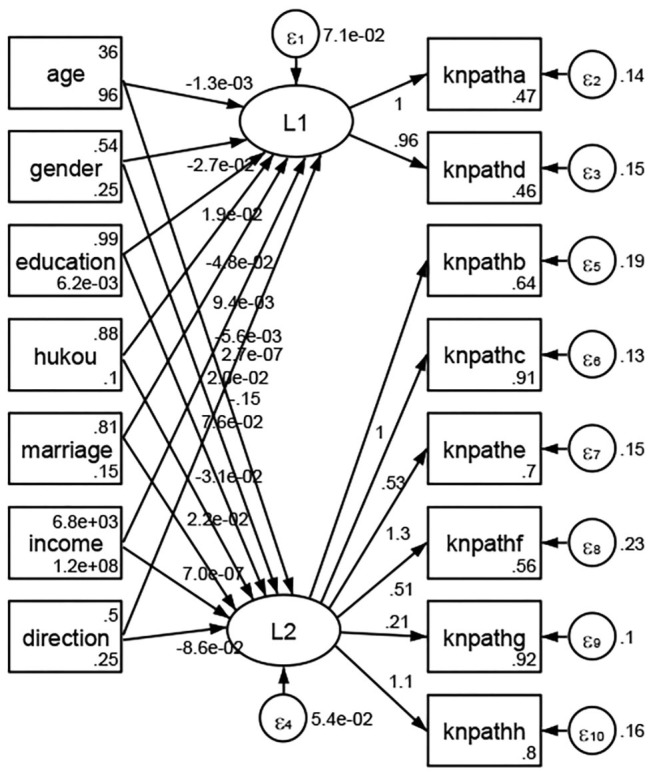
Hunan.

**Figure 43:**
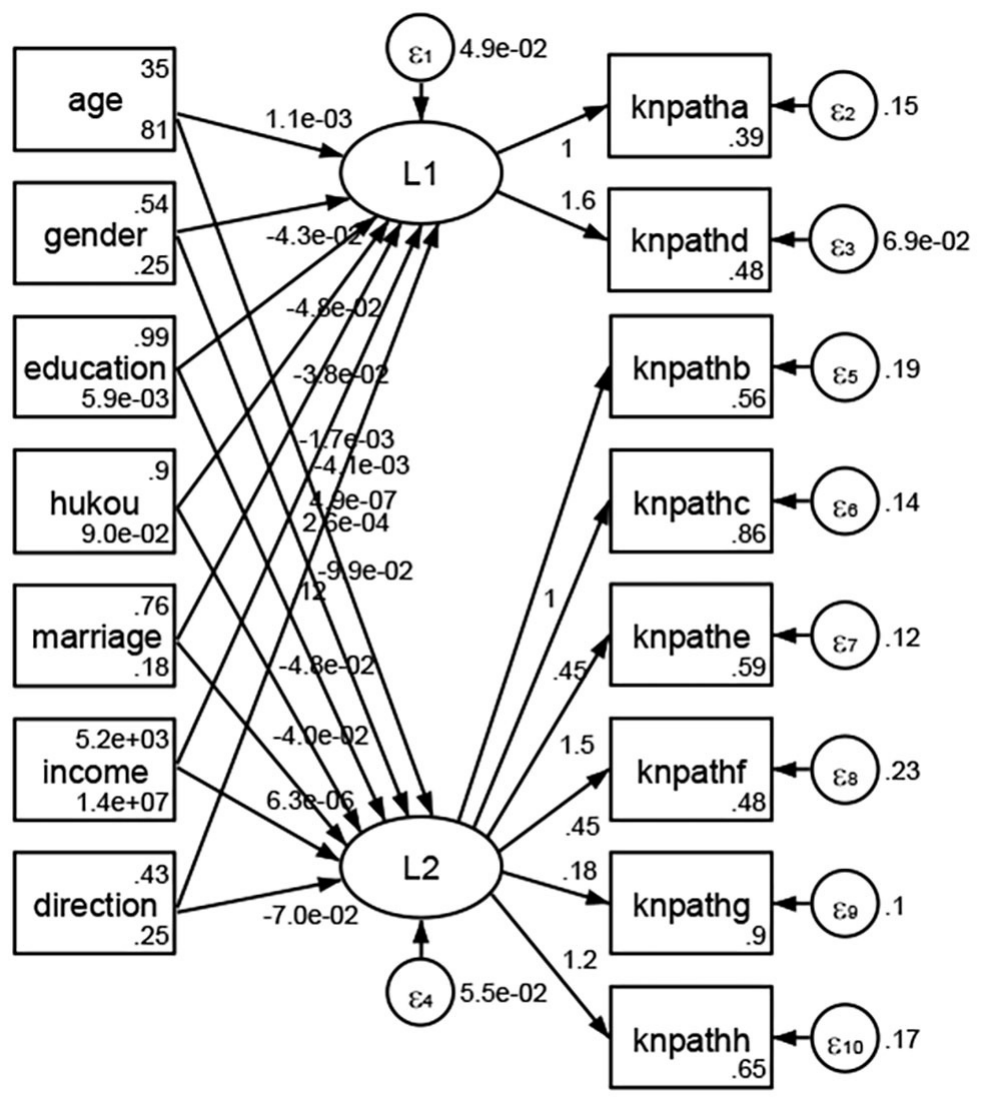
Guangxi.

**Figure 44:**
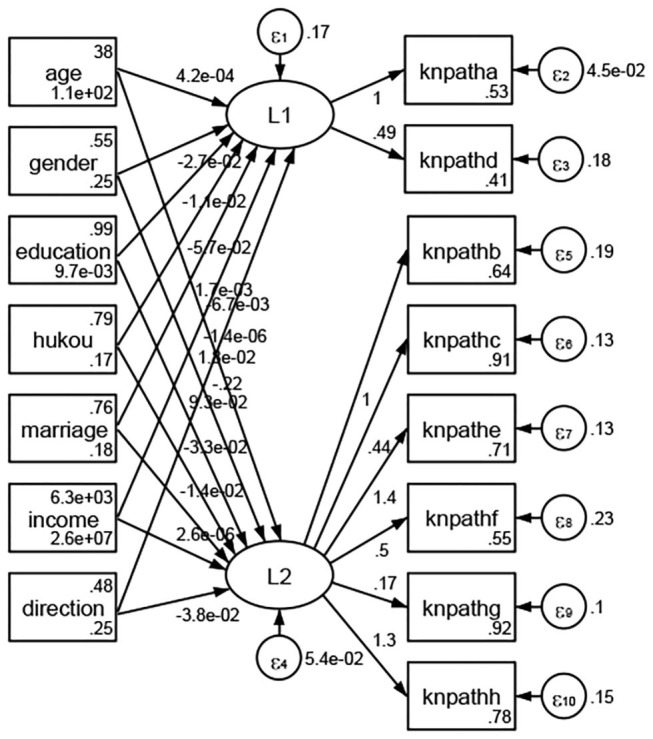
Chongqing.

**Figure 45:**
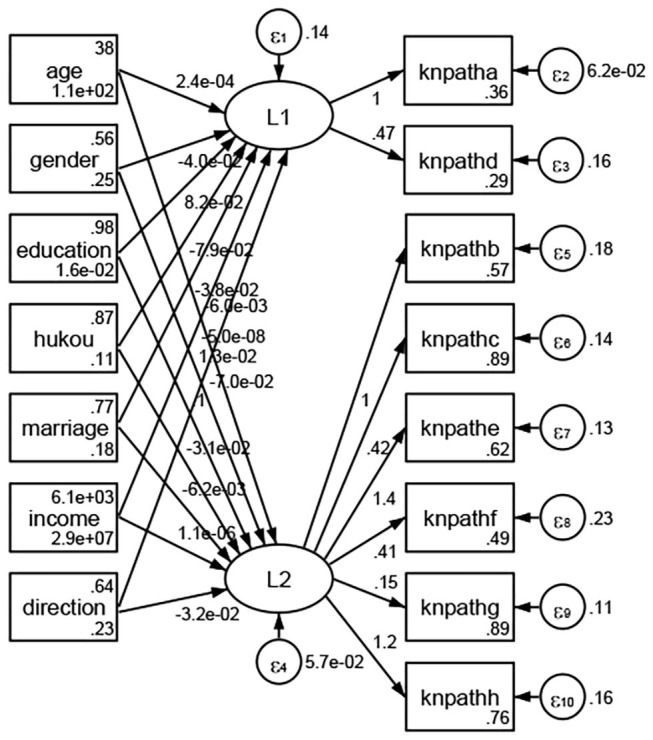
Sichuan.

**Figure 46:**
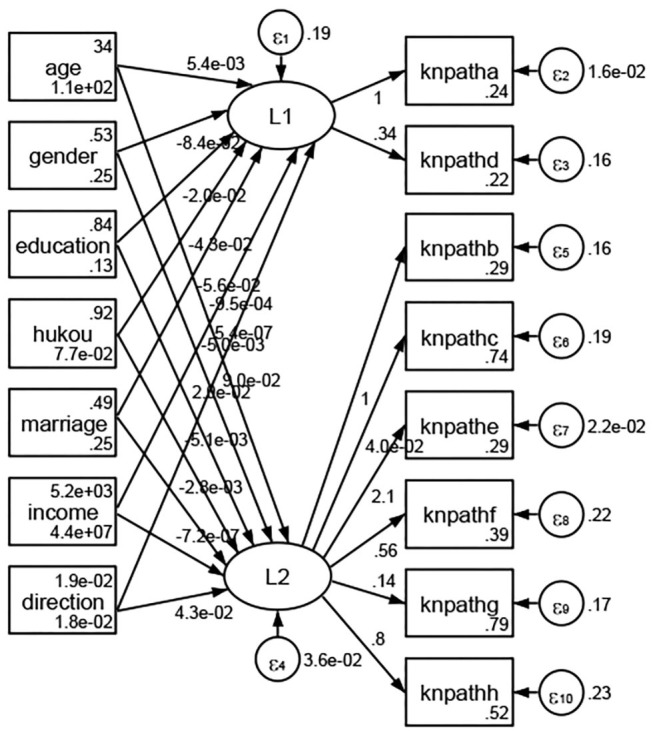
Tibet.

**Figure 47:**
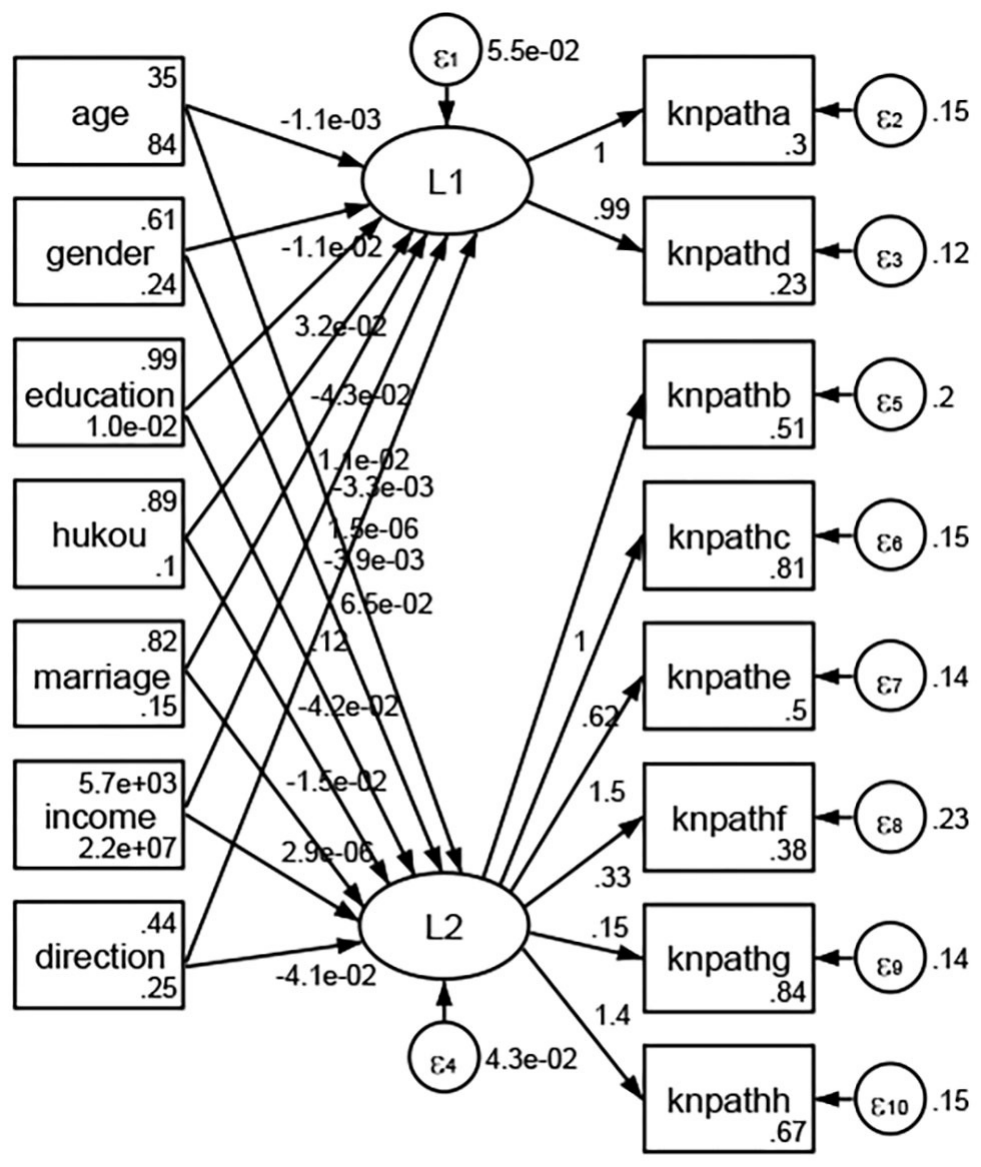
Shannxi.

**Figure 48:**
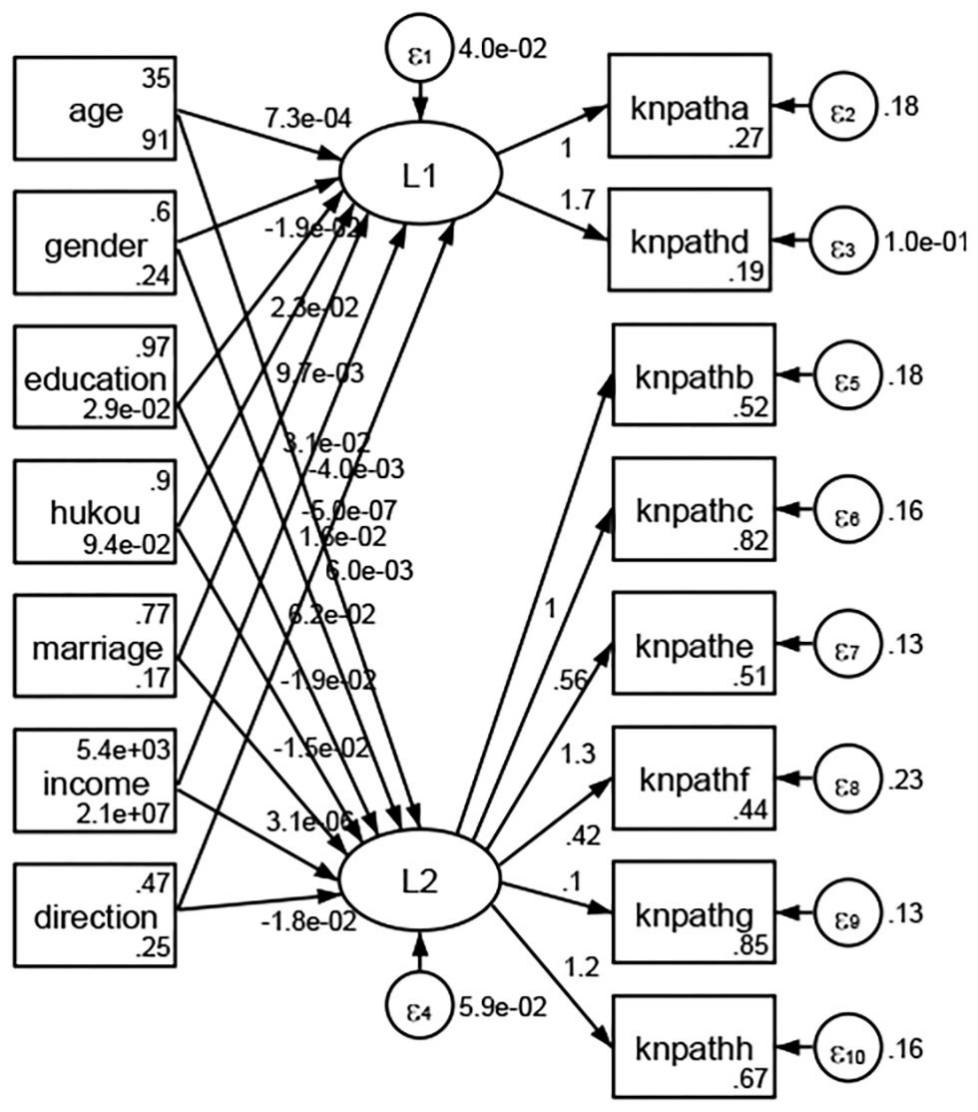
Gansu.

**Figure 49:**
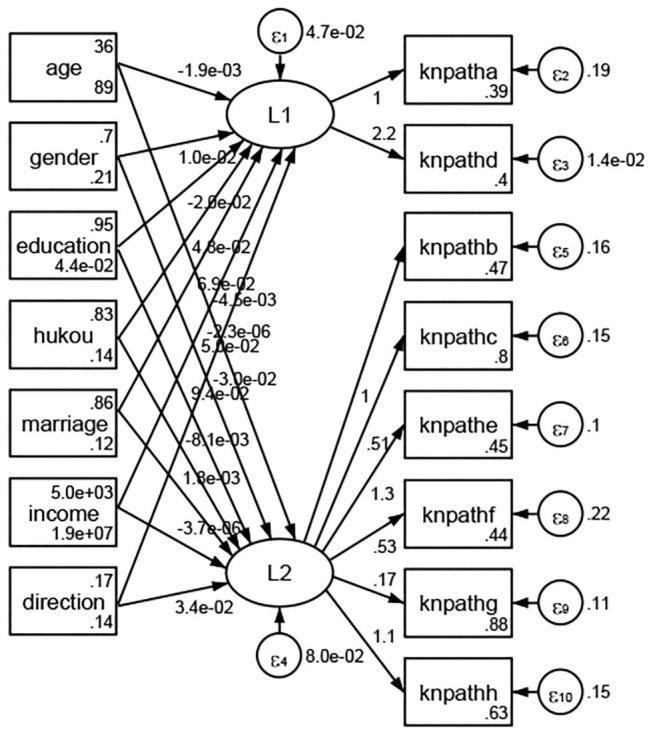
Ningxia.

**Figure 50:**
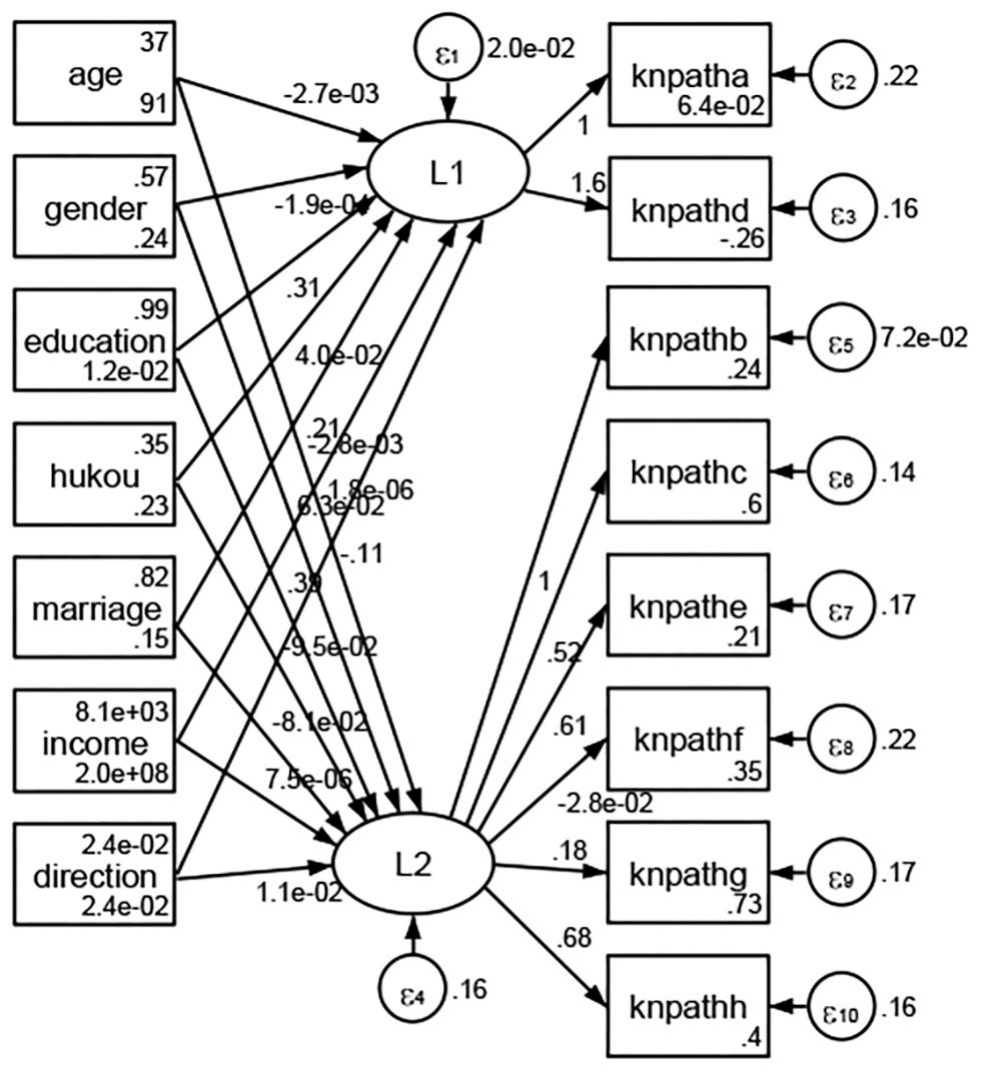
XPCC.

Except Beijing, Tianjin, Shanxi,Inner Mongolia, Liaoning, Jilin, Heilongjiang, Shanghai, Guangdong, Hainan, Guizhou, Yunan, Gansu, Qinghai, Xinjiang, and XPCC, Figures 35–50 showed RMSEA values in total sample (RMSEA = 0.060), Hebei (RMSEA = 0.070), Jiangsu (RMSEA = 0.063), Fujian (RMSEA = 0.060), Jiangxi (RMSEA = 0.060), Henan (RMSEA = 0.060), Hubei (RMSEA 0 = 0.063), Hunan (RMSEA = 0.062), Guangxi (RMSEA = 0.075), Chongqing (RMSEA = 0.066), Sichuan (RMSEA = 0.061), Tibet (RMSEA = 0.078), Shannxi (RMSEA = 0.055), Gansu (RMSEA = 0.060), Ningxia (RMSEA = 0.070), and XPCC (RMSEA = 0.095) were <0.1. Thus, provincial imbalance was reflected by the associations between geodemographic factors and methods of access to health knowledge.

### Association Between Economic Zones and Health Records

See Table 4. Regarding health records, the coefficients of Yangtze River Delta, Bohai Rim, and other region decreased in order. Thus, the zone imbalance of health records was reflected by the association between economic zones and health records.

**Table 4:** Association between economic zones and health records.

	**Health records**
Yangtze river delta	0.204^***^ (0.006)
Bohai rim	0.603^***^ (0.015)
Other region	1.059^***^ (0.023)
Pearl river delta	0.671^***^ (0.013)
Pseudo *R^2^*	0.0485
Observations	138327

*Standard deviations are in parentheses*.

*** *represented 1%. Pearl River Delta was referred. Yangtze River Delta consists of south central Jiangsu, Shanghai, and northeast Zhejiang. Bohai Rim is composed of Beijing, Tianjin, Liaoning, Hebei, Shanxi, Shandong, and Inner Mongolia. Pearl River Delta refers to Guangdong, Hongkong, and Macao*.

### Association Between Economic Regions and Social Medical Insurance

See Table 5. Regarding NRCMS (new rural cooperative medical scheme), the coefficients of central China, western China, and Northeast China decreased in order. Regarding CMIURR (cooperative medical insurance for urban and rural residents), the coefficients of central China, western China, and northeast China decreased in order. Regarding URBMI (urban resident based basic medical insurance), the coefficients of central China, western China, and northeast China decreased in order. Regarding UEBMI (urban employee-based basic medical insurance), the coefficients of central China, western China, and northeast China decreased in order. Regarding FMT (free medical treatment), the coefficients of central China, northeast China, and eastern China decreased in order. Thus, regional differences were reflected by the associations between economic regions and social medical insurance.

**Table 5:** Regional differences in the social medical insurance coverage.

	**NRCMS**	**CMIURR**	**URBMI**	**UEBMI**	**FMT**
Central China	2.316^***^ (0.041)	0.915^**^ (0.040)	1.635^***^ (0.054)	0.282^***^ (0.006)	0.541^**^ (0.164)
Western China	1.371^***^ (0.017)	2.685^***^ (0.074)	1.931^***^ (0.052)	0.311^***^ (0.005)	1.245 (0.226)
Northeast China	0.804^***^ (0.017)	0.719^***^ (0.052)	3.528^***^ (0.130)	0.357^***^ (0.012)	0.109^**^ (0.110)
Eastern China	1.914^***^ (0.015)	0.030^***^ (0.001)	0.036^***^ (0.001)	0.352^***^ (0.003)	0.001^***^ (0.0001)
Pseudo *R^2^*	0.0155	0.0306	0.0189	0.0509	0.0090
Observations	162,345	161,864	161,914	162,165	161,840

*Standard deviations are in parentheses*.

*** and

** *represent 1 and 5%. Central China includes Shanxi, Anhui, Jiangxi, Henan, Hubei, and Hunan. Western China includes Inner Mongolia, Guangxi, Chongqing, Sichuan, Guizhou, Yunan, Tibet, Shannxi, Gansu, Qinghai, Ningxia, and Xinjiang. Northeast China includes Liaoning, Jilin, and Heilongjiang. East China includes Beijing, Tianjin, Hebei, Shanghai, Jiangsu, Zhejiang, Fujian, Shandong, Guangdong, and Hainan*.

## Discussion

This study reflected that the majority of the sample was educated and married with interprovince and intercity migration. Their establishment proportion of health records was low. Regarding social medical insurance, NRCMS (new rural cooperative medical scheme) coverage rate was the highest in the insurance system. Regarding types of access to health knowledge, kntypeb (nutritional health knowledge), kntypec (reproduction and contraception/eugenics knowledge), kntypee (smoking control knowledge), and kntypeh (STD/AIDS knowledge) accounted for more than half of the sample. Regarding methods of access to health knowledge, more than half of the sample utilized knpathc (radio/TV programmes), knpathg (bulletin board), and knpathh (SMS/WeChat) to access health knowledge. There were provincial, economic zones, and regional differences in the public health services for the floating population. There were geographic difference and provincial difference in the associations between geodemographic factors and public health services. Thus, there were geographical imbalances for the floating population to access comprehensive, affordable, and effective health care services.

The findings in this study were in accord with early studies which reported that public health services were not sufficient among Chinese floating population. For example, current research indicated that the comprehensive behavior intervention (19) was necessary to strengthen due to high prevalent rate of pulmonary tuberculosis (20), high risk factors for congenital heart defect (21), and obese (22) among the floating population. A substantial research provided potential and possible explanations for the necessary improvement of medical services in the floating population. For instance, a review indicated that the major hypertension problem of floating population was caused by their unhealthy lifestyle (drinking) and deficient disease management (23). With low household healthcare expenditure (24) and health insurance coverage (25), most of them lacked health knowledge (26), reproductive health knowledge and skills (27), and HIV-related sexual knowledge (28). Simultaneously, there existed a certain percentage of self-treatment behavior for rural floating populations when they felt uncomfortable or sick (29). Hence, public health services should be improved for the floating population.

The key point is that inadequate funds explained the reasons for the uneven distribution of essential public health services. Although implementation of the health reform since 2009 received a commendation from several scholars (30), another study indicated that the average investment of 3.97 USD per capita in the National Essential Public Health Services Package issued in 2009 was lower than the amount needed to meet its running costs among common residents (31). Another study explained that the reason for inequality in public health services, and the inefficiency of the fiscal transfer payment system, was Chinese local officials' fanatical pursuit of local economic growth in the vast majority of provinces (32). A study concluded that outsourced institutions provided by the primary medical institutions performed worse than public institutions led by the Chinese government in terms of efficiency and quality (33). In fact, despite developed cities such as Zhuhai, a cross-sectional study found government funds for basic public health services could not compensate for the actual costs under the constraints of a limited budget (34).

Other factors with minor importance also were documented in the early research. Early studies reported that market-oriented financing reforms of public health services (35), clear and detailed protocols (36), structural social capital (37), household registration (38), satisfaction with the attitude of primary health care workers (39), and social integration (40) were responsible for an increased utilization of local public health services among domestic migrants in China.

### Strength and Limitations

The strengths of this study were to focus on the relationship between geodemographic factors and access to public health services. To my best knowledge, this was the first study to report the associations between geodemographic factors and access to public health services among the Chinese floating population. The current study used multistage clustering random sampling method and large sample size, which increases the generalizability of the findings.

There were two limitations. First, cross-sectional data cannot reflect dynamic relationships. The cross-sectional design limited the ability to get more valuable conclusions related to causality. Second, samples of Beijing, Tianjin, Shanghai, Hongkong, Macao, and Taiwan were less than 100. Compared to large sample of the other provincial-level units, the statistical results in the provincial-level units with small samples lacked significance. Especially, significant associations of geodemographic factors with types of access to health knowledge and methods of access to health knowledge could be confirmed in part of the provincial-level units (provincial-level units with significant outcomes/total provincial-level units: 30/35 and 16/35).

### Policy Implications

The main findings of this study were regional imbalance in the case of establishment rate of health records, social medical insurance ratio, access to health knowledge ratio, and methods of access to health knowledge ratio. Regional differences were associated with social medical insurance and the methods of health knowledge attainment. The findings of this study were a good angle from which to recommend the national plans and policies related to the floating population.

For the purpose of boosting the health and well-being of the Chinese floating population, public health services for migrant populations should be improved. With respect to policy improvement and its future directions in China's floating population, this study indicated biased investment should be provided to public health services in unprivileged regions, areas neglected by policy, and economically undeveloped areas. Additional compensatory investment should be introduced to ensure public health services to remain adequately funded. The local governments of provincial-level units should launch targeted policies such as health knowledge education for the floating population. In order to promote the equalization of essential public health services, social organizations and community should undertake the responsibility in social support for the internal migrants. Meanwhile, comprehensive measures to improve capacity of primary health care sectors must be emphasized. Practically, recommendations to enable migrants to obtain basic public health services include abolishing the separation of local and migrant household registration, increasing local government's fiscal capacity, and improving multi-sector cooperation with health information systems.

## Conclusions

In conclusion, access to public health services is low and varied widely across provincial-level units. Significant associations between geodemographic factors and access to public health services among Chinese floating population were confirmed in part of the country. Due to its unintended consequences, geographic imbalance of public health services should be paid attention by local policy-designers. Particularly, the targeted improvement of the effectiveness of health management needs an increase of local funds.

## Data Availability Statement

Publicly available datasets were analyzed for this study. These can be found here: The survey dataset used in this study could be obtained on the website: http://www.chinaldrk.org.cn/wjw/#/home.

## Author's Note

MG is head of the International Issues Center and Family Issues Center at Xuchang University. He is interested in health care service, health change, and quality of life of migrants and elders in modern China.

## Author Contributions

MG designed the study, performed the statistical analysis, and completed the original version.

## Conflict of Interest

The authors declare that the research was conducted in the absence of any commercial or financial relationships that could be construed as a potential conflict of interest.

## Abbreviations

XPCC: Xinjiang Production and Construction Corps
NRCMS: new rural cooperative medical scheme
CMIURR: Cooperative medical insurance for urban and rural residents
URBMI: urban resident based basic medical insurance
UEBMI: urban employee-based basic medical insurance
FMT: free medical treatment
knpatha: access to health knowledge with lecture
knpathb: access to health knowledge with books/magazine/CD
knpathc: access to health knowledge with radio/TV programmes
knpathd: access to health knowledge with face-to-face consultation
knpathe: access to health knowledge with online medical consultation
knpathf: access to health knowledge with community advocacy
knpathg: access to health knowledge with bulletin board
knpathh: access to health knowledge with SMS/WeChat
kntypea: access to occupational disease prevention knowledge
kntypeb: access to nutritional health knowledge
kntypec: access to reproduction and contraception/eugenics knowledge
kntyped: access to chronic disease prevention knowledge
kntypee: access to smoking control knowledge
kntypef: access to mental disorders knowledge
kntypeg: access to tuberculosis knowledge
kntypeh: access to STD/AIDS knowledge
kntypei: access to other infectious diseases knowledge
ERHR: establishment rate of health records
SMIR: social medical insurance ratio
IHKR: inaccess health knowledge ratio
MIHKR: methods of inaccess to health knowledge ratio
ERHR: persons with health records/total sample
SMIR: persons with social medical insurance/ total sample
IHKR: persons with inaccess to health knowledge/ total sample
MIHKR: persons with inaccess methods of access to health knowledge/ total sample
MIMIC: Multiple indicator multiple cause model
PCA: Principal component analysis
KMO: Kaiser-Meyer-Olkin
RMSEA: root mean square error of approximation
IRB: Institutional Review Board.
